# ECBD: European chemical biology database

**DOI:** 10.1093/nar/gkae904

**Published:** 2024-10-23

**Authors:** Ctibor Škuta, Tomáš Müller, Milan Voršilák, Martin Popr, Trevor Epp, Katholiki E Skopelitou, Federica Rossella, Katja Herzog, Bahne Stechmann, Philip Gribbon, Petr Bartůněk

**Affiliations:** CZ-OPENSCREEN: National Infrastructure for Chemical Biology, Institute of Molecular Genetics of the Czech Academy of Sciences, Vídeňská 1083, Prague 14220, Czech Republic; CZ-OPENSCREEN: National Infrastructure for Chemical Biology, Institute of Molecular Genetics of the Czech Academy of Sciences, Vídeňská 1083, Prague 14220, Czech Republic; CZ-OPENSCREEN: National Infrastructure for Chemical Biology, Institute of Molecular Genetics of the Czech Academy of Sciences, Vídeňská 1083, Prague 14220, Czech Republic; CZ-OPENSCREEN: National Infrastructure for Chemical Biology, Institute of Molecular Genetics of the Czech Academy of Sciences, Vídeňská 1083, Prague 14220, Czech Republic; CZ-OPENSCREEN: National Infrastructure for Chemical Biology, Institute of Molecular Genetics of the Czech Academy of Sciences, Vídeňská 1083, Prague 14220, Czech Republic; EU-OPENSCREEN ERIC, Robert-Rössle-Str. 10, Berlin 13125, Germany; EU-OPENSCREEN ERIC, Robert-Rössle-Str. 10, Berlin 13125, Germany; Fraunhofer-Institute for Translational Medicine and Pharmacology ITMP, Schnackenburgallee 114, Hamburg 22525, Germany; EU-OPENSCREEN ERIC, Robert-Rössle-Str. 10, Berlin 13125, Germany; EU-OPENSCREEN ERIC, Robert-Rössle-Str. 10, Berlin 13125, Germany; CZ-OPENSCREEN: National Infrastructure for Chemical Biology, Institute of Molecular Genetics of the Czech Academy of Sciences, Vídeňská 1083, Prague 14220, Czech Republic

## Abstract

The European Chemical Biology Database (ECBD, https://ecbd.eu) serves as the central repository for data generated by the EU-OPENSCREEN research infrastructure consortium. It is developed according to FAIR principles, which emphasize findability, accessibility, interoperability and reusability of data. This data is made available to the scientific community following open access principles. The ECBD stores both positive and negative results from the entire chemical biology project pipeline, including data from primary or counter-screening assays. The assays utilize a defined and diverse library of over 107 000 compounds, the annotations of which are continuously enriched by external user supported screening projects and by internal EU-OPENSCREEN bioprofiling efforts. These compounds were screened in 89 currently deposited datasets (assays), with 48 already being publicly accessible, while the remaining will be published after a publication embargo period of up to 3 years. Together these datasets encompass ∼4.3 million experimental data points. All public data within ECBD can be accessed through its user interface, API or by database dump under the CC-BY 4.0 license.

## Graphical Abstract

**Graphical Abstract:**
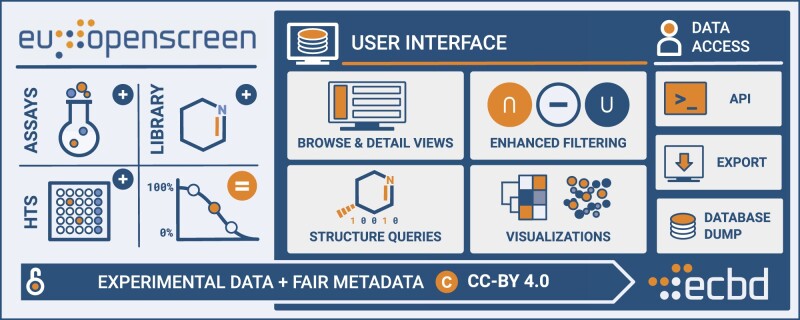

## Introduction

Modern approaches to early drug discovery and the related discipline of chemical biology increasingly rely on the ability to access, integrate and analyze diverse large datasets, comprising structural, clinical and bioassay data, amongst others (1–3). These data inform decision-making related to hit selection, compound optimization, probe design or lead development; their effective use being enabled by the adoption of FAIR (findable, accessible, interoperable and reusable) data principles (4). The generation and dissemination of FAIR data is one of the main tenets of EU-OPENSCREEN, which was established in 2018.

As a large publicly funded research infrastructure consortium, EU-OPENSCREEN provides molecular screening and chemistry support to external users, enabling them to conduct large-scale discovery projects in chemical biology and early drug discovery. The consortium consists of 31 partner sites, comprising compound screening platforms, medicinal chemistry groups, cheminformatics and chemoproteomics specialists, across Europe (https://www.eu-openscreen.eu/about/partner-sites.html). These partner sites jointly use EU-OPENSCREEN’s collection of compounds, incorporating (i) the European Chemical Biology Library (ECBL) (5), which contains over 100 000 commercially sourced small molecules; (ii) the European Academic Compound Library (EACL), which contains an increasing number of compounds (∼5700 currently) provided by chemists in both European and non-European countries; and (iii) the European Fragment Screening Library (EFSL; >1000 compounds) designed to facilitate biophysical-based hit-finding and optimization activities through a direct substructural alignment with the ECBL (6). These collections are available to external scientists for collaborative discovery and development projects exclusively at EU-OPENSCREEN screening partner sites. These compound screening resources significantly contribute towards removing bottlenecks in early-phase drug discovery and accelerating the development of novel chemical probes in basic research.

EU-OPENSCREEN ensures the FAIRness of all data generated within their supported projects. As such, the complete datasets are made available to the scientific community via the European Chemical Biology Database (ECBD), including both positive (active) and negative (inactive) activity data. Holding data of a similar nature, ECBD plays a similar role to the PubChem Bioassay database (7), storing bioactivity information from high-throughput and medicinal chemistry studies. In contrast with other related sources, such as ChEMBL (8), BindingDB (9), ZINC (10), Probes & Drugs (11), Guide To Pharmacology (12), DrugBank (13) or DrugCentral (14), which focus mainly on confirmed (validated) bioactivity data extracted from scientific publications and patents, ECBD contains data from the entire chemical biology project pipeline, including the primary assay as well as counter-screening data. Compared to PubChem Bioassay, the largest public chemical biology data resource by far (as of July 2024, containing 1671 253 assays and almost 300 million bioactivities), ECBD currently holds a smaller amount of data with a realistic outlook of having several hundreds of assays and tens of millions of data points in the upcoming years. In contrast, ECBD’s two main assets reside in (i) the standardized, quality-focused data acquisition process and (ii) the full-deck screening principle.

Being released quite recently, ECBD can from the very beginning take advantage of an established FAIR approach to achieve standardized and detailed data description, utilizing common formats and ontologies in the field (see Table 1 and SI), some of which were not available 10 years ago. This in combination with the defined assay standards across EU-OPENSCREEN screening sites should ensure high data and metadata quality of the datasets released into the public domain.

**Table 1.** The list of ontologies employed for the metadata description during the data submission

Ontology	Description	URL
BioAssay ontology (BAO)	Describes chemical biology screening assays and their results including high-throughput screening (HTS) data	http://bioassayontology.org/
Units of measurement ontology (UO)	An ontology of units of measurements	https://github.com/bio-ontology-research-group/unit-ontology
Cellosaurus (CSS)	A knowledge resource on cell lines	https://www.cellosaurus.org/
BRENDA tissue ontology (BTO)	A structured controlled vocabulary for the source of an enzyme	https://www.brenda-enzymes.org/
Gene ontology (GO)	A logical structure describing the full complexity of the biology	https://geneontology.org/
ChEMBL (gene ontology) Protein target slim (CPTS)	A broad categorization of the biology of 90% of the protein targets using just 300 high-level, informative GO terms	https://jbiomedsem.biomedcentral.com/articles/10.1186/s13326-016-0102-0
NCBI taxonomy (NCBIT)	A curated classification and nomenclature for all of the organisms in the public sequence databases	https://www.ncbi.nlm.nih.gov/taxonomy/
Reactome pathway hierarchy (RPH)	A genome-wide, hierarchical organization of Reactome pathways	https://reactome.org/

The full-deck screening principle dictates that only one or more full compound libraries (see ‘Data model’ and ‘Compounds’ sections) must be employed within a project, ensuring full chemogenomic/physicochemical property coverage of the screened chemical space (or sub-space, depending on the employed library/libraries). Furthermore, EU-OPENSCREEN itself performs a panel of bioprofiling screens (solubility, autofluorescence, absorbance, reactive oxygen species, luciferase inhibition, antibacterial/antifungal activity, cell viability and bacterial/fungal growth inhibition) for all compounds within ECBL and EACL to increase the detail of their description and informationally support their evaluation within other projects.

These two factors, along with the availability of both positive and negative results, make a strong foundation for machine learning tasks, increasingly being integrated into modern drug discovery workflows, and which can greatly benefit from curated, high-quality and consistently built datasets, that are thoroughly described with controlled vocabularies and ontologies (15).

## Data acquisition

All experimental data in ECBD are generated within the EU-OPENSCREEN screening network utilizing the infrastructure’s compound libraries. To mitigate the often observed low reproducibility of results acquired from high-throughput screening (HTS) campaigns (16,17) and to enhance their re-usability and interoperability, the process of data acquisition is controlled, including (i) the quality control (QC) of the compounds by the EU-OPENSCREEN’s central compound management facility (CCMF) and the sourcing of the library batches to the screening sites; (ii) the compliance of these screening sites with common assay standards adopted by EU-OPENSCREEN; and (iii) the data upload into the ECBD performed by trained members of the screening site where the assay was performed.

### Quality control and sourcing of compounds

To assure the correctness of experimental data, all EU-OPENSCREEN compounds are submitted to QC analysis using an ultra-high-performance liquid chromatography instrument with three detector options: (i) Mass spectroscopy: Single quadrupole or time-of-flight to confirm the identity by comparing the expected and found mass of the molecule, (ii) diode array detector to determine the purity by quantifying the detected peaks and (iii) evaporative light scattering detector to determine the purity of compounds that do not provide any signal in the UV/Vis chromatogram. After the raw data analysis, the QC results and reports are deposited in the ECBD and are available on their respective compound detail pages (see ‘Web interface’ section). Details of the method are presented in the Supplementary File S1.

For the EFSL, the purity and identity of fragments is assessed by nuclear magnetic resonance (6) with ECBD providing links to the reports stored at the Biological Magnetic Resonance Bank (18).

The compounds are plated in DMSO (dimethyl sulfoxide) at 10 mM concentration on 384-well plates and distributed by the CCMF to the respective partner sites for use in assays. Plates are shipped on dry ice with precise parcel tracking and batch control, further contributing to the quality assurance of the screening data in ECBD.

### Compound screening

Assay quality criteria are applied across the network of partner sites based on the EU-OPENSCREEN HTS QC general guidelines (Supplementary File S2). For biochemical assays, reagents (e.g. proteins, enzymes, buffers) have to be stable in the experimental conditions tested for the entire duration of the experiment, and the signal stability proven before embarking on a screen. For cell-based assays, the quality and integrity of cell lines should be proven through short tandem repeat profiling (19). Where possible, a minimum Z′ factor cut off is applied to confirm the required signal window between ‘active’ and ‘inactive’ compounds; otherwise, data variability is reported using the coefficient of variation. After assay development and adaptation, screening of the 5000 compound EU-OPENSCREEN pilot library (see ‘Data model’ and ‘Compounds’ sections) serves to establish the assay protocol, data evaluation, estimation of hit rate and test for robustness of the assay against compound-induced measurement artefacts (e.g. caused by cytotoxicity, autofluorescence or aggregation).

### Data upload and description

For data upload, a dedicated interactive web-based form was developed, accessible only by trained EU-OPENSCREEN partner site members. While the experimental data are uploaded as simple data files (in most cases containing only compound IDs, concentrations and values measured within the experiment), most descriptive metadata terms are selected from underlying ontologies, controlled vocabularies or other classification systems (Table 1) (20–28). For the sake of simplicity, we will use the term ‘ontology’/‘ontological’ further in the paper as a simplified umbrella-term for various commonly employed classification systems and description frameworks despite many of them not being ontologies *per se*, e.g. the NCBI Taxonomy (27) or Reactome pathway hierarchy (28).

**Figure 1.**
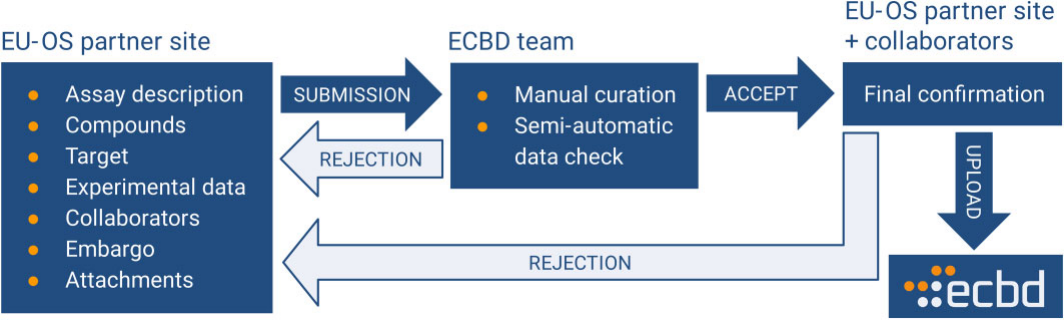
The data upload workflow. First, the experimental data are submitted along with their metadata description, attachments, assigned collaborators and set embargo. Next, the submission is curated by the ECBD team, both manually and automatically, where possible. For the submission to be accepted, it has to be finally confirmed by the submitter as well as assigned collaborators, and only then all data are uploaded into the database. If the submission is rejected in the second or third step, it is returned to the submitter to make the necessary changes.

To ensure metadata consistency across different experiments and data submitters, the ontological fields (i.e. fields that should be filled with a term from an ontology) are tightly connected to a specific branch of the ontology to narrow the list of possible options that can be used (also to give the user a better idea about the meaning of the field, since identical terms can be used differently by different people/teams/laboratories). Being aware of ontology imperfections, data submitters are allowed to use a custom term if they don’t find a suitable one or actively label the field as ‘not applicable’ in the context of their assay. The data and metadata are further curated (Figure 1) by the ECBD team and then finally confirmed by the assay (project) collaborators (external assay providers) before the actual upload into the database. If the submitter chooses not to use an embargo (up to 36 months), the data become instantly public after the upload. Otherwise, only the submitter and project collaborators have access to the data during the embargo period and the data are automatically published once this embargo period expires.

## Data model

The ECBD works with three main interconnected data types: compounds, assays and targets. These main data types can be browsed, filtered and exported through the ECBD UI (see ‘Web interface’ section), employing standardized data formats commonly used for their description and identification not only in ECBD but across the entire chemical biology data resource environment. At the same time, each of these data objects can be uniquely identified and referenced through their EU-OPENSCREEN persistent identifier, EOS ID.

### Compounds

The full compound set in ECBD comprises individual libraries (ECBL, EFSL and EACL) with ECBL containing differently purposed sub-libraries: diversity, bioactive and nuisance (serving as a quasi-control compound set, representing various assay interference compound classes). Also, several libraries are either a representative subset or a combination of others (Table 2). The primary identifier of a compound is its InChIkey (29,30) and each InChIkey is also connected with its EOS persistent identifier (in a few cases where compounds from different libraries overlap, a single structure can be associated with multiple EOS identifiers), starting from EOS1. Both of these identifiers can be used to reference the compound (i.e. https://ecbd.eu/compound/EOS100077 and https://ecbd.eu/compound/ICCFXXDUYSPKOL-UHFFFAOYSA-N leads to the same compound).

**Table 2.** The list of all EU-OPENSCREEN compound libraries and sub-libraries with their compound count and a brief description

Sub-library (library)	Count	Description
**Diversity (ECBL)**	**98 560**	Large diversity library selected by EU-OPENSCREEN sites
**Representative diverse set (ECBL)**	**2 464**	A representative subset of the diversity library
**Bioactives (ECBL)**	**2 464**	Custom bioactives library with emphasis on wide target coverage
**Nuisance (ECBL)**	**88**	A representative set of nuisance compounds in biological screening
**Pilot library (ECBL)**	**5 016**	Combination of the (i) representative diverse set, (ii) bioactives and (iii) nuisance libraries
**Fragments (EFSL)**	**1 056**	Fragment library selected by EU-OPENSCREEN sites
**Mini 88 fragments (EFSL)**	**88**	A representative subset of the fragments library
**Academic compounds (EACL)**	**5 280**	Compounds submitted to EU-OPENSCREEN by academic institutions
**Total**	**107 414**	-

Besides primary identifiers, each compound is described by its name and CAS registry number (if available), additional structural representations (SMILES, InChI, MolFile and formula) and properties connected to the structure itself: basic physicochemical properties [including Lipinski’s rule-of-five (RO5) (31)], PAINS structural alerts (32,33), and links to other databases [harvested through UniChem (34)]. Furthermore, each compound is supplied with its QC parameters and a report.

### Targets

ECBD recognizes several types of targets: protein, protein complex, nucleic acid, cell-line, pathway, tissue, organism and no target (e.g. for physicochemical property endpoints). Each type is separately identified by a commonly used persistent identifier or an ontology term (with a persistent Internationalized Resource Identifier). The target can be further described by its context—additional information based on the target type, e.g. the cell line used within a ligand-binding assay or the biological pathway regulated through the assay’s primary protein target.

- Single proteins and protein complexes are primarily identified by the UniProt ID (35) or IDs, respectively, (the system also allows for the use of gene names and ChEMBL target names/IDs which can be useful especially for multi-component targets).
- Nucleic acids are primarily identified by their GenBank ID (36). If this is not available, they can be defined by their reference to another resource (ID/URL), type (DNA, RNA, mRNA, etc.), the organism of origin based on the NCBI taxonomy (27) and the sequence (optional).
- Cell lines are described by the combination of their type (primary or immortalized) and the specific cell line selected from Cellosaurus ontology (22). Cell lines can be used both as the primary defined target as well as an additional term describing the target context.
- Pathways are defined based on the term from the Reactome pathway hierarchy (28). The pathway can also be used as an additional term to describe the target context, for example of a protein target.
- Tissues are defined based on the term from the BRENDA tissue ontology (23) and can also be used as an additional descriptor of the target’s context.
- Organisms are defined through the NCBI taxonomy (25). Apart from being a primary target of the experiment, an organism can also be used to describe the context of the primary target, and separately, an organism is also defined on the global level of the assay (assay organism field).

### Assays

Each assay represents a single experiment performed with (i) one or more full compound libraries (see Table 2), mainly in the case of primary assays (generally, assays performed with a larger number of samples in duplicate/triplicate in one or two concentrations to identify ‘interesting’ compounds, so-called primary hits, for more rigorous testing, i.e. their confirmation) or (ii) primary hit selection, mainly in the case of confirmatory or counter-screening assays.

During the data submission, the assay is described in detail by its depositor utilizing (i) free-text input for the overall assay/protocol description and the activity determination approach (i.e. how were the hits/active compounds selected); (ii) ontological description of the assay’s main parameters (such as assay stage, assay format, physical detection method, etc.), assay’s biological target and its context (e.g. a single protein target tested in a specific cell-line perturbing a specific pathway, and an assay’s readout description (such as concentration/time units and a specific value type, e.g. Z-score, percent inhibition, etc.); (iii) optional file attachments (such as assay protocol, assay/project presentation, data visualizations and others).

The full list of employed ontologies and specific ontological fields with their basic description can be found in Table 1 and Supplementary File S1, respectively.

### Endpoints and results

In addition to the main data types, each assay contains (i) one or more data endpoints and (ii) a result for each individual tested compound.

The endpoint represents a quantitative measure of the compound’s response (assay’s readout) or its normalized/transformed value. For example, the assay can contain (a) raw luminescence values (first endpoint) together with their z-score values (second endpoint); (b) two endpoints with measurements using different wavelengths accompanied by their ratio as the third endpoint; or (c) percentage inhibition for different concentrations as one endpoint and the dose-response curve parameters as the second one.

The result is the final mandatory text evaluation of the compound’s outcome (‘activity call’) within an assay which has to be defined by the data submitter. For activity-based assays, the options are: active, inconclusive, inactive, error and undefined, however, if these options are not suitable in the assay context (e.g. for physicochemical property measurements) the submitter can define his/her own consistent result classification system (e.g. for solubility measurements, high, medium and low solubility). There’s always only one result value for each compound-assay pair supported by one or more endpoint values. The endpoint/result values can also be supplemented with the reference samples’ values (e.g. reference compounds or empty wells) and their definition (e.g. reference type, name and structure).

## Data content

**Figure 2.**
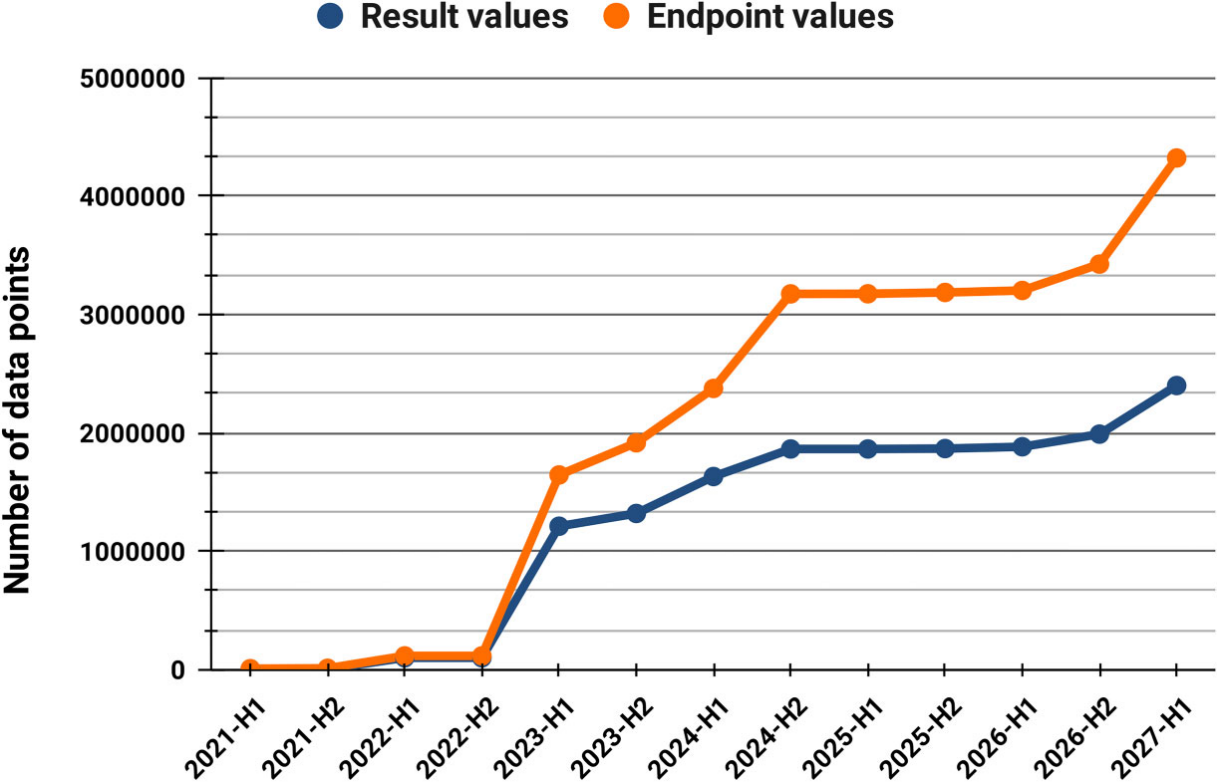
The evolution of endpoint and result values stored in ECBD (H1—first half of the year, H2—second half of the year). The first submission was made in H1 2021, while the latest submission, currently under embargo, will be published in H1 2027.

In total, 89 data submissions have been made into ECBD with 48 public as of August 2024. These comprise ∼2.5 million public endpoint values (∼4.3 million in total) and ∼1.7 million public result values (∼2.4 million in total). Based on the embargo periods, all data currently stored in the database will be available in the first half of 2027 at the latest. The current count of the main ECBD data types can be found in Table 3 accompanied by the progress chart representing the evolution of endpoint and result values in time (Figure 2).

**Table 3.** The current count of the main ECBD data types with the associated endpoint and result values

**Data type**	**Count**	**Count including non-public**
**Compounds**	107 414	107 414
**Assays**	48	89
**Targets**	20	33
**Endpoint values**	2 463 519	4 321 105
**Result values**	1 684 759	2 398 232

Currently, the majority of data values originate from assays labelled as primary (*n* = 30) and confirmatory (*n* = 16) with the following bioassay types: 17 functional phenotypic, 14 binding, 8 functional target-based, 5 functional and 4 physicochemical. The most prevalent target assay organism is human (*n* = 22), and the most prevalent specific bioassay type is cell-growth assay (*n* = 19). See Supplementary File S3 for a detailed list of assays and their parameters. As of August 2024, there were 9042 hits identified within primary assays with 2112 (23.4%) later confirmed.

### Connection to external data resources

To enhance the interconnection and re-usability of the data, ECBD actively registers and shares some of its public datasets with other resources. Confirmatory screens with validated bioactivity values are uploaded to ChEMBL, a key data bioactive resource in chemical biology (first sets being public from ChEMBL ver. 34, accessible at https://www.ebi.ac.uk/chembl/g/#search_results/documents/query=ecbd). Suitable datasets are further shared within the international projects EU-OPENSCREEN is a participant in, such as the BY-COVID project (https://by-covid.org/) (37) and the EOSC4CANCER project (https://eosc4cancer.eu/). Furthermore, the whole ECBD compound library is registered and awaiting approval within the EBI’s UniChem service (34), a non-redundant database maintaining small molecule cross references between different chemistry resources. The bioactive subset of ECBL is registered and can be accessed at the Probes & Drugs portal (10), a hub for the integration of high-quality bioactive compound sets (accessible at https://www.probes-drugs.org/compounds/standardized#compoundset=353@AND). In the near future, most of the public datasets will be uploaded to the PubChem Bioassay database not only to enhance their re-usability, but also to reduce the fragmentation of available data in the field.

## Web interface

The web user interface is the most common way of accessing ECBD data. It is designed to be user-friendly and work well on both desktop and mobile devices. The whole application, including the database, is hosted within the CESNET (Czech Education and Scientific NETwork, https://www.cesnet.cz/en/) infrastructure with Python (https://www.python.org/) (Django framework, https://www.djangoproject.com/) and JavaScript (Vue.js framework, https://vuejs.org/) being the main employed programming languages, combined with the PostgreSQL database system (https://www.postgresql.org/) and the RDKit cheminformatics framework (https://www.rdkit.org/).

### Browse and detail views

**Figure 3.**
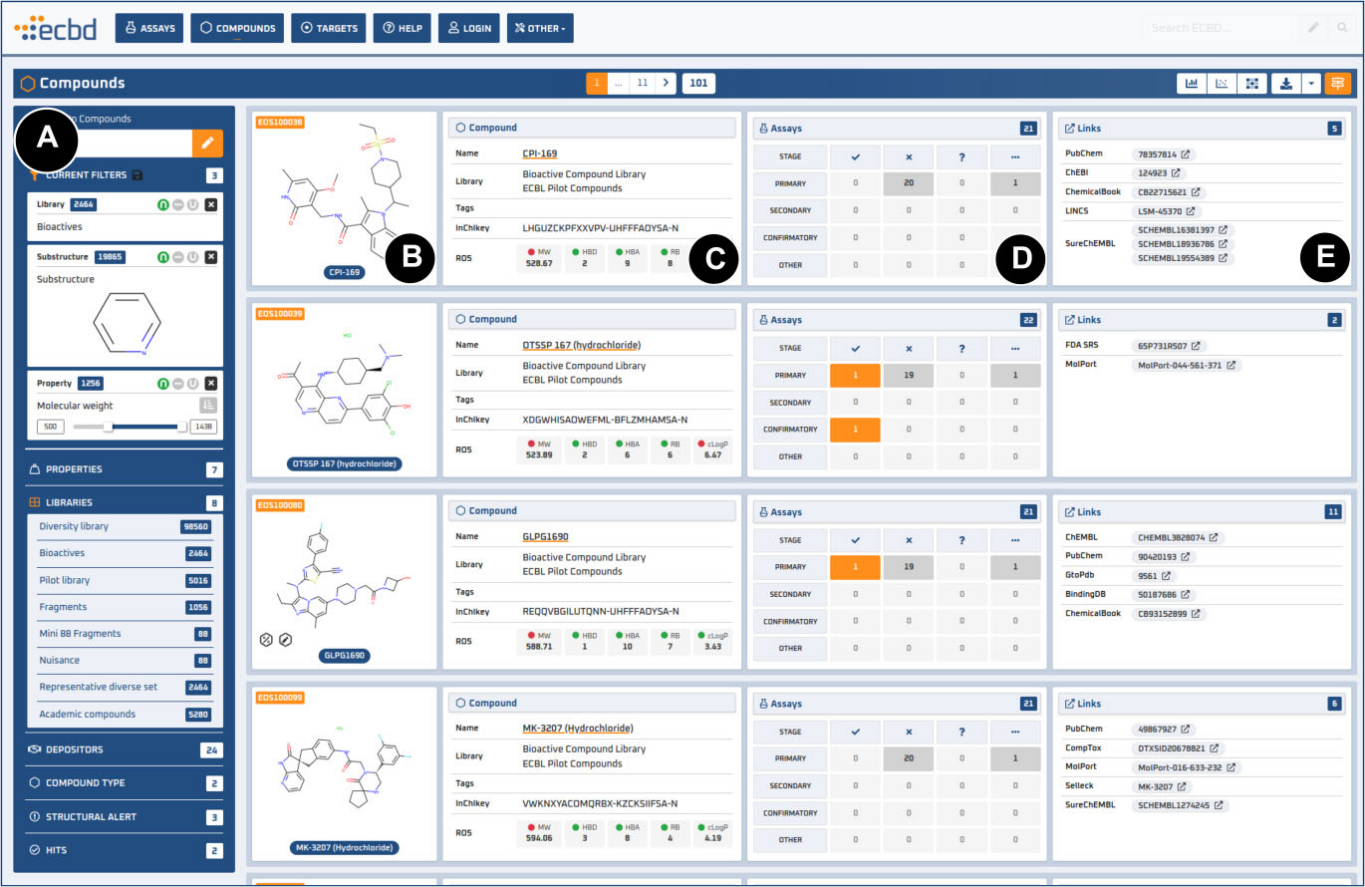
Compound browse view with the left navigation bar (**A**) and the representation of a compound divided into four sections (B–E). (A) The navigation bar contains (from top to bottom) a text search field enhanced with chemical structure editor (pencil button on the right), three currently applied filters: the intersection of compound library(s), substructure and physicochemical property filters, and below that a set of ready-to-use filters divided into categories. (B) The compound’s structure (with a highlighted substructure based on the used substructure filter), EOS ID, a name and action buttons (appearing on hover) to invoke similarity search and open the structure within the structure editor. (C) General information including main identifiers, compound libraries, tags and the Lipinski’s RO5 physicochemical properties. (D) The summary of the compound’s assay results. (E) Links to external databases.

**Figure 4.**
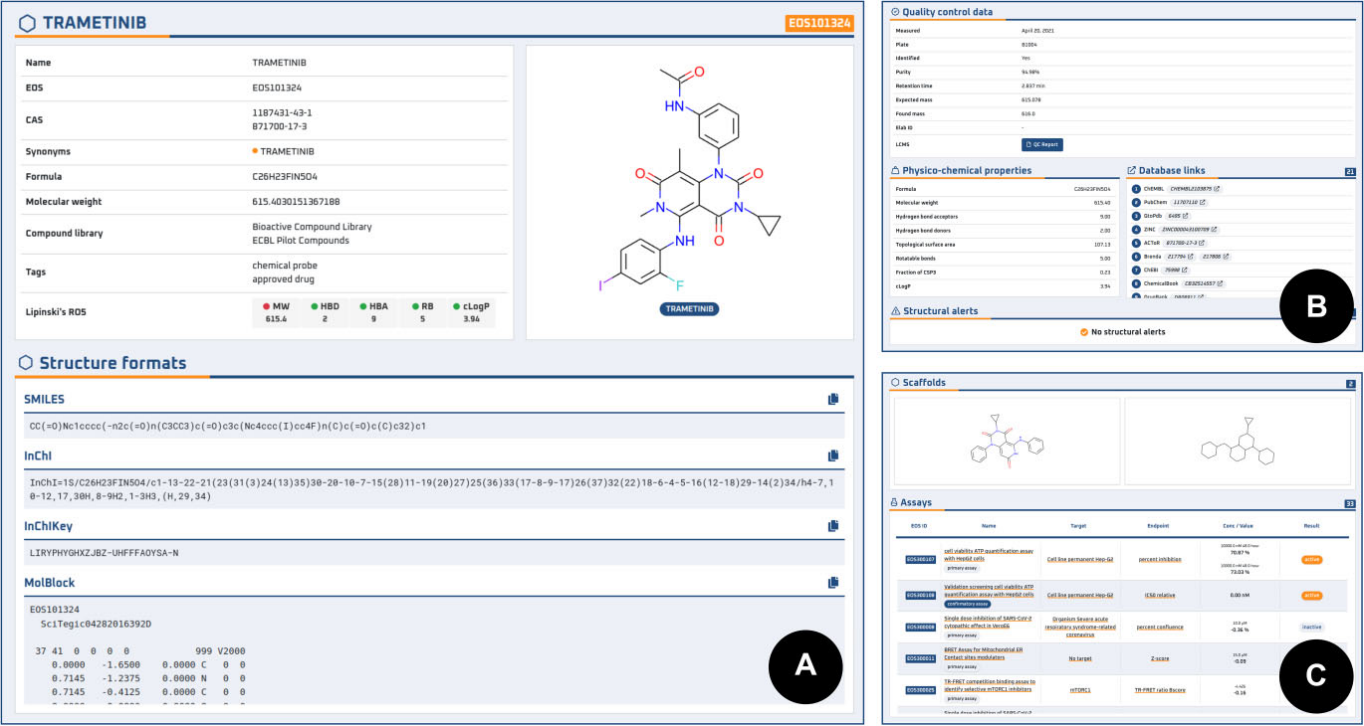
Compound detail view containing (from top to bottom) (A) general information including identifiers, tags, compound libraries, RO5 properties and structure image with different text formats (SMILES, InChI, InChIkey and MolBlock); (B) QC data with the PDF report, basic physicochemical properties, links to external databases and structural alerts (if any); (C) scaffolds - Bemis-Murcko (BM) and generic BM, and detailed assay results.

The interface provides two common approaches for accessing the main data types (assays, compounds and targets): a browse and a detailed view. In the browse view, all objects are listed with their main attributes (e.g. title, type, ID, image). The user can either browse through individual pages or narrow the scope of displayed data using the search input with filters on data attributes (Figure 3). In the detailed view, all data associated with a single object are summarized on a single page (Figure 4). Each view is represented by a separate web page with its permanent web address, allowing it to be easily linked or referenced.

**Figure 5.**
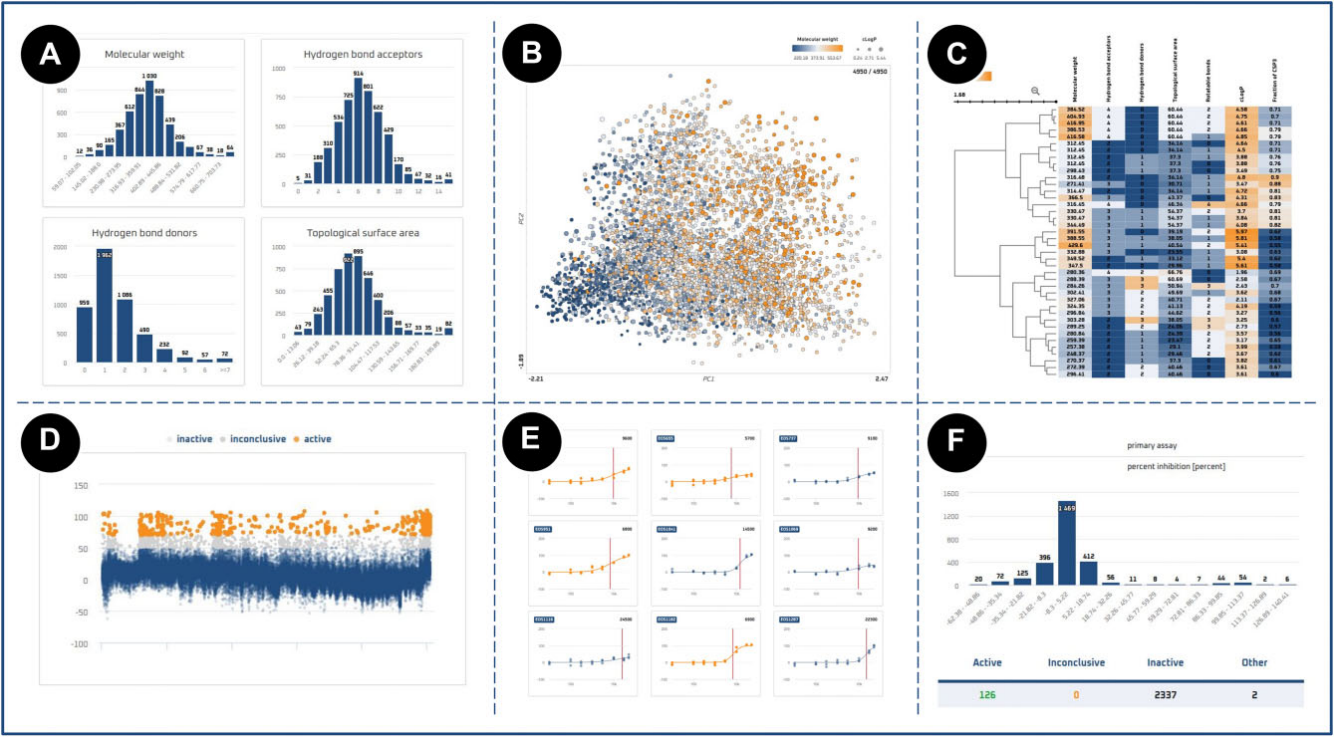
Data visualizations used in ECBD. (A) Bar chart distribution of compounds’ physicochemical properties. (B) Chemical space visualization of a selected compound set. (C) Cluster heatmap of a selected compound set. (D) Scatterplot visualization of assay’s endpoint values colored based on the compound’s result within the assay. (E) Dose-response curve visualization based on the supplied curve parameters and measured experimental values. (F) Bar chart distribution of endpoint values on the target detail page.

Both views employ visualizations appropriate for the display of data in a given context. For basic visualizations, such as the activity distribution of ligands within an assay/target or physicochemical property distribution of a filtered compound set, bar charts and scatter plots are utilized; for more complex relationships, such as nearest-neighbour associations of compounds within chemical space or hierarchical data structures, specialized libraries, ChemSpace.js (https://openscreen.cz/software/chemspace) and InCHlib (Interactive Cluster Heatmap Library) (38), are integrated (Figure 5).

### Search and filtering

Basic data search is implemented in a Google-like fashion, i.e. all data can be searched from a simple persistent (i.e. always available) single field using an autocomplete functionality. During the text search, a list of prioritized terms, accompanied by their object types (e.g. compound, target, assay, etc.), is suggested. In addition to the text search, chemical structure queries enable searching for compounds based on their identity, similarity, substructure or superstructure. Chemical structure queries are implemented through an integrated chemical structure editor [Ketcher (39)], but users can write/copy-paste the query structure to the search text field as well. Structure-based queries are implemented through the RDKit cheminformatics database cartridge (https://www.rdkit.org/docs/Cartridge.html) which enables millions of compounds to be searched within milliseconds. Search results are sorted based on a query logic, e.g. according to compounds’ similarity to the query structure.

In addition to the search functionality, ECBD browse views feature a powerful filtering system enabling users to query various properties of the browsed objects. The system allows for the construction of not only single filter queries but also of more complex–multi-conditional–ones. The basic unit is a single filter, which can be of different types. Generally, a filter represents a subset of the full dataset that is applied based on its associated logical Boolean operator, represented by its mathematical symbol (**∩** = intersection, **−** = difference, **∪** = union). The result of a used filter depends on the selected operator and the dataset on its input. For the first selected filter, the input set is naturally equal to the full dataset (i.e. for the intersection, the result is equal to the objects represented by the filter, for difference, to the full set without the objects represented by the filter, and for union, to the full set, since any set united with its subset is equal to the former). Multiple filters can then be chained together into a logical expression. In that case, each filter is applied subsequently to the set resulting from the preceding one. If a change occurs anywhere in the expression (e.g. one of the Boolean operators changes), the result is instantly re-evaluated. Since the data query is reflected in the URL, it can be easily used to share the current filtered dataset. Registered users are also allowed to save their data queries under an arbitrary name for later re-use.

## Data access

ECBD provides users with the most common approaches of data access suitable for different needs. First is the access through the ECBD’s web interface, where data can be inspected in real-time and where exports (*.csv*,*.xlsx*and*.json*) can be performed for all data objects (compounds, targets or assays) or any of their subset containing their primary identifiers with annotations. Individual compound sets can also be downloaded from the Downloads section (https://ecbd.eu/download) through the web interface in *.csv*,*.xlsx and .sdf* format. For fast data access on local computers/servers, the PostgreSQL database dump with all public data is also available in the Downloads section. For users accessing the data programmatically, either from a custom script or from an application that can utilize the application programming interface (API), such as KNIME (40), ECBD provides a REST API accessible at https://ecbd.eu/api/ (see Supplementary File S1 for API examples). All public data are licensed under the Create Commons Attribution 4.0 International License (CC BY 4.0), i.e. the users have the freedom to reuse the data in any way, if they acknowledge EU-OPENSCREEN and the use of ECBD and add no additional restrictions on the data.

## Summary

ECBD is a central hub for experimental data generated within the EU-OPENSCREEN infrastructure. Its adherence to standardized procedures, emphasis on QC and detailed FAIR assay description results in the availability of reliable, high-quality datasets that are reproducible and well-suited as training data for virtual screening. That, in combination with the requirement for full-library screens and the EU-OPENSCREEN bioprofiling effort, will contribute to more detailed characterization of chemical space defined by the EU-OPENSCREEN compound libraries with each new screen. Currently, there are 89 datasets uploaded in the database with 48 already publicly available and the rest to be published upon expiry of their embargo period. The data can be acquired in various formats and be freely used under the CC BY 4.0 license.

The ECBD will expand in the near future to accommodate data from recent EU-OPENSCREEN initiatives. Soon, EU-OPENSCREEN is planning to perform cell painting experiments (41) with the entire compound library. This effort will generate one of the largest publicly available, *in vitro* cellular image datasets, and the ECBD will include the original data as well as the resulting cellular fingerprints. Another dataset addressed to the issue of reproducibility of experimental screening data will be made available from an internal EU-OPENSCREEN ring-testing effort, where all screening sites will perform the same panel of representative screening experiments. As with currently ECBD-housed data, the results of these efforts will be accompanied by detailed protocols and ontological description of the data, in accordance with FAIR principles.

## Supplementary Material

gkae904_Supplemental_Files

## Data Availability

The ECBD is freely available at https://ecbd.eu. Public data stored in ECBD are made available under the CC BY 4.0 license (https://creativecommons.org/licenses/by/4.0/).
