# Correction to ‘ECBD: European chemical biology database’

**DOI:** 10.1093/nar/gkaf026

**Published:** 2025-01-24

This is a correction to: Ctibor Škuta, Tomáš Müller, Milan Voršilák, Martin Popr, Trevor Epp, Katholiki E Skopelitou, Federica Rossella, Katja Herzog, Bahne Stechmann, Philip Gribbon, Petr Bartůněk, ECBD: European chemical biology database, *Nucleic Acids Research*, Volume 53, Issue D1, 6 January 2025, Pages D1383–D1392, https://doi.org/10.1093/nar/gkae904

In the originally published version of this manuscript, author Katja Herzog was inadvertently omitted from the author list. This error has been corrected.

