# Supplementary material for: ECBD: European chemical biology database
Source: Nucleic Acids Res. 2024 Oct 23;53(D1):D1383–92. doi: 10.1093/nar/gkae904 (PMC11701612; doi:10.1093/nar/gkae904)
Supplement: gkae904_Supplemental_Files [file gkae904_supplemental_files.zip › supplementary_file_1.pdf]

Supplementary file 1 for ECBD: European Chemical Biology Database

Ctibor Škuta[1,*], Tomáš Müller[1], Milan Voršilák[1], Martin Popr[1], Trevor Epp[1], Katholiki E. Skopelitou[2], Federica Rossella[2], Bahne Stechmann[2], Philip Gribbon[2,*] and Petr Bartůněk[1,*]

[1] CZ-OPENSCREEN: National Infrastructure for Chemical Biology, Institute of Molecular Genetics of the Czech Academy of Sciences, Prague, 14220, Czech Republic
[2] EU-OPENSCREEN ERIC, Berlin, 13125, Germany

* To whom correspondence should be addressed. Email: skutac@img.cas.cz

Correspondence may also be addressed to philip.gribbon@eu-openscreen.eu and bartunek@img.cas.cz.

Compounds quality control (QC)

The entire library was controlled for identity and purity. The QC procedure for all compounds is as follows: a 2 µL sample of each 10 mM stock of compound dissolved in DMSO, was diluted to 0.02 mM in 50:50 water/acetonitrile for injection into a UPLC. The sample was separated using a C18 chromatographic column using a gradient of acetonitrile and ammonium formate buffer, with 0.05% formic acid. The total run time is 2.4 minutes.

Chemicals are detected using a DAD detector at 230 nm, and the molecular mass measured in a single quadrupole mass detector or in a time-of-flight detector. While the sample is separated and analyzed, a second, identical column is regenerated with a washing method using buffer and acetonitrile, having the column ready for the next analytical run. This approach greatly increases the analysis rate and minimizes the carry-over effect.

Raw data were then analyzed using Mass Hunter 10.0 (Agilent Technologies, Santa Clara, CA, USA) and Virscidian Studio Reviewer Pro (Virscidian Inc, Cary, NC, USA) software solution for generation of individual .csv and .pdf data to be uploaded into the database.

Workflows developed with KNIME are also used to create lists of files, check errors and to create worklists for preparing plates for data analysis.

We observed that about 15 % of samples do not present any detectable UV signal, due to the lack of chromophores. In such cases, samples were analyzed with an Evaporative Electronic Light Scattering Detector (ELSD), a less-sensitive but universal detector based on the refraction of nebulized particles. In this case, the purity of the compound is based on the AUC of the ELSD chromatogram.

The purity and identity of fragments compounds have been assessed through NMR as described by Jalecias et al., 2004 10.1039/d3md00724c. Briefly, deuterated-DMSO stock solutions were dissolved in 25 mM sodium phosphate buffer, 150 mM NaCl, 5% d6-DMSO, and 10 µM sodium trimethylsilylpropanesulfonate (DSS) at pH 7.5 and 1 H-NMR spectra were acquired.

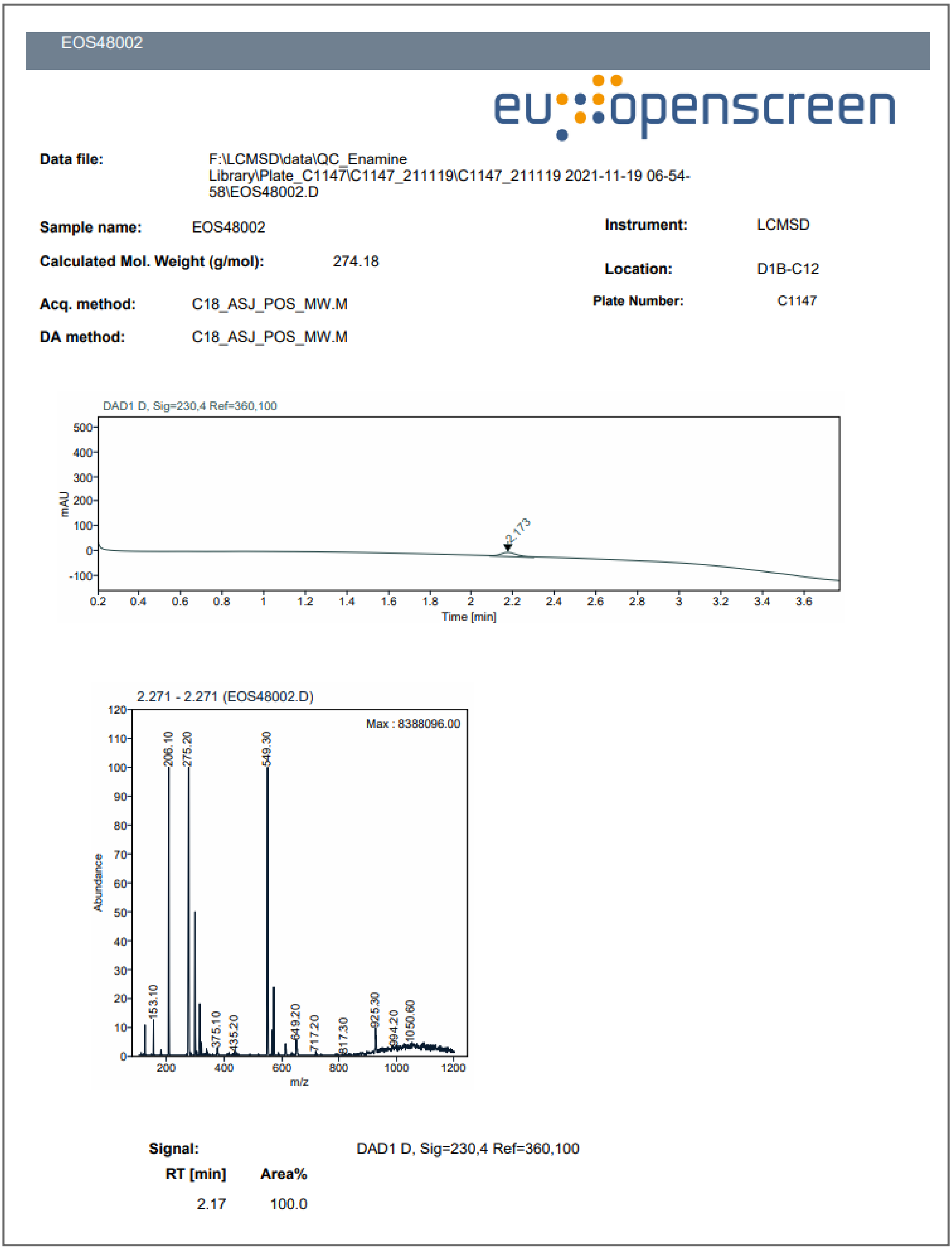

EOS48002

Data file: F:\LCMSD\data\QC_Enamine Library\Plate_C1147\C1147_211119\C1147_211119 2021-11-19 06-54-58\EOS48002.D

Sample name: EOS48002

Instrument: LCMSD

Calculated Mol. Weight (g/mol): 274.18

Location: D1B-C12

Acq. method: C18_ASJ_POS_MW.M

Plate Number: C1147

DA method: C18_ASJ_POS_MW.M

Signal: DAD1 D, Sig=230,4 Ref=360,100

RT [min]	Area%
2.17	100.0

Figure 1. An example of the one-page QC report in PDF format stored as an attachment of the Compound object in the ECBD. The top part contains sample and method details, below the DAD chromatogram and mass spectra of the selected peaks are displayed. At the bottom of the page, the AUC results of the found peaks are presented.

Ontological assay description

Table 1. The list of all ontological fields used for the assay (dataset) description during the upload into ECBD.

Field	Ontology	Branch (IRI)	Example
**Assay stage**	BAO	assay screening campaign stage (BAO_0000029)	primary assay
**Bioassay type**	BAO	bioassay type (BAO_0000008)	binding type
**Bioassay**	BAO	bioassay (BAO_0000015)	kinase activity assay
**Bioassay setting**	BAO	experimental setting (BAO_0020005)	in vitro
**Assay format**	BAO	assay format (BAO_0000019)	cell-based format
**Assay design**	BAO	assay design method (BAO_0002202)	luciferase induction
**Assay supporting method**	BAO	assay supporting method (BAO_0002429)	photoaffinity labelling
**Physical detection method**	BAO	physical detection method (BAO_0000035)	alpha screen
**Detection instrument**	BAO	detection instrument (BAO_0000697)	Operetta
**Assay organism**	NCBIT	-	Homo sapiens
**Cellular component**	GO	cellular component (GO_0005575)	cytoplasm
**Concentration unit**	UO	concentration unit (UO_0000051)	nanomolar
**Time unit**	UO	time unit (UO_0000003)	second
**Additional tags**	BAO	-	fungal meningitis

Web interface

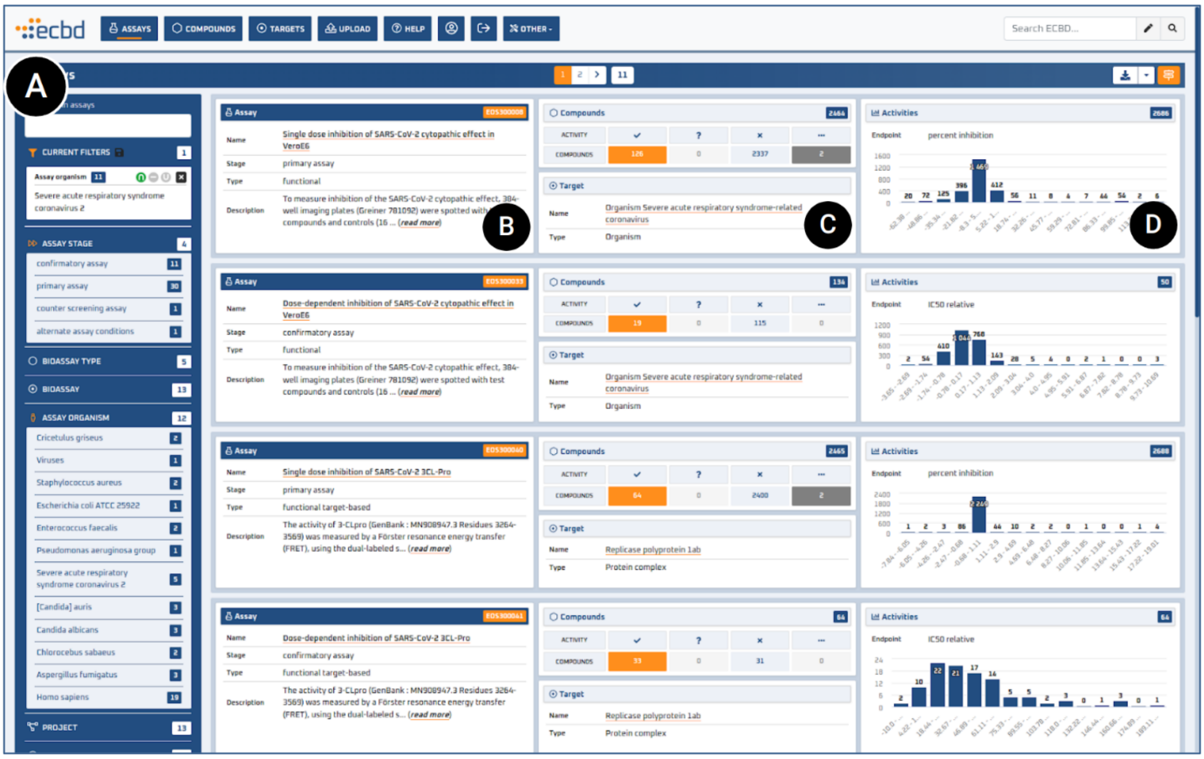

Figure 2. Assay browse view with the left navigation bar (A) and the representation of an assay divided into 3 sections (B - D). A) The navigation containing (from top to bottom) a text search field, 1 currently applied filter for an assay organism. B) The assay's general information - EOS ID, a name, stage, type and description. C) The number of used compounds with their result within the assay (C - top); The assay target with its type (C - bottom). D) The experimental values distribution bar chart.

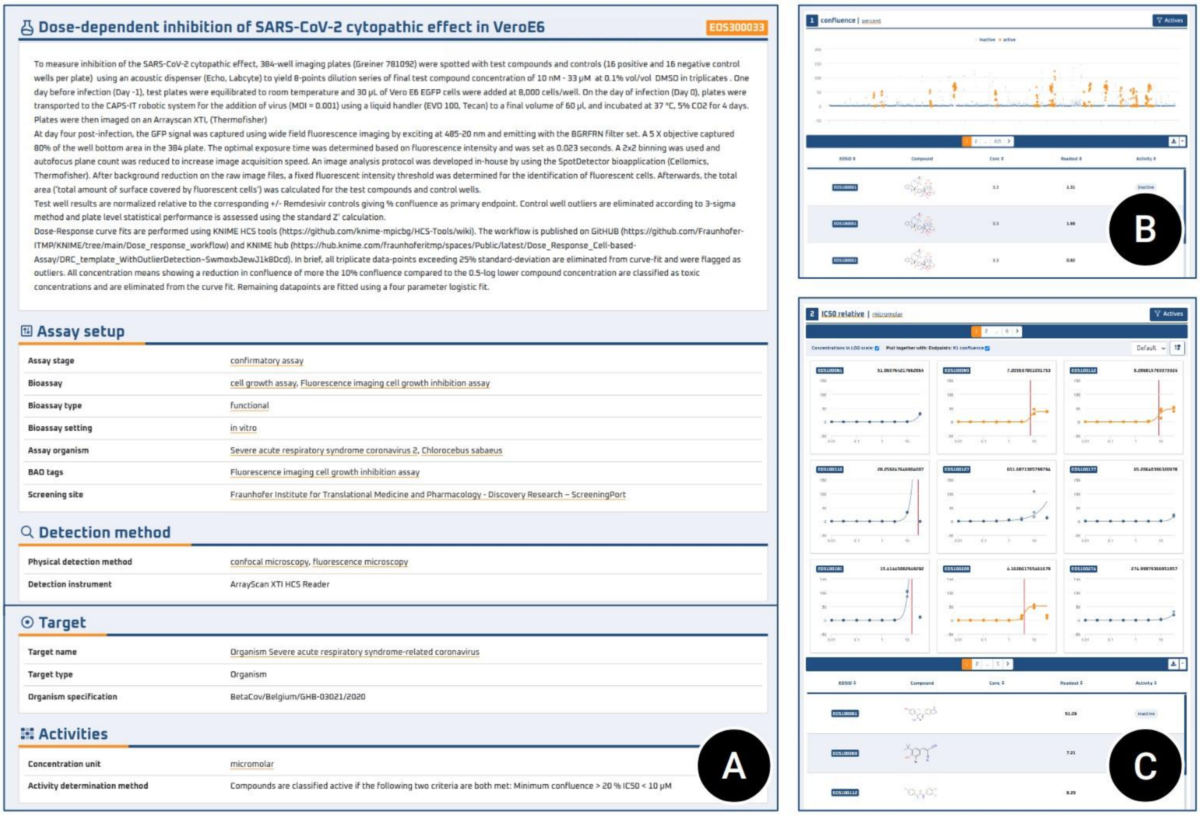

Figure 3. Assay detail view containing (from top to bottom) A) General information including text description and ontological description of the assay setup, detection method, target and activities; B) The colour-coded endpoint data (measured values) scatterplot based on the compounds' result within the assay with the data table; C) The dose-response curves with the data table.

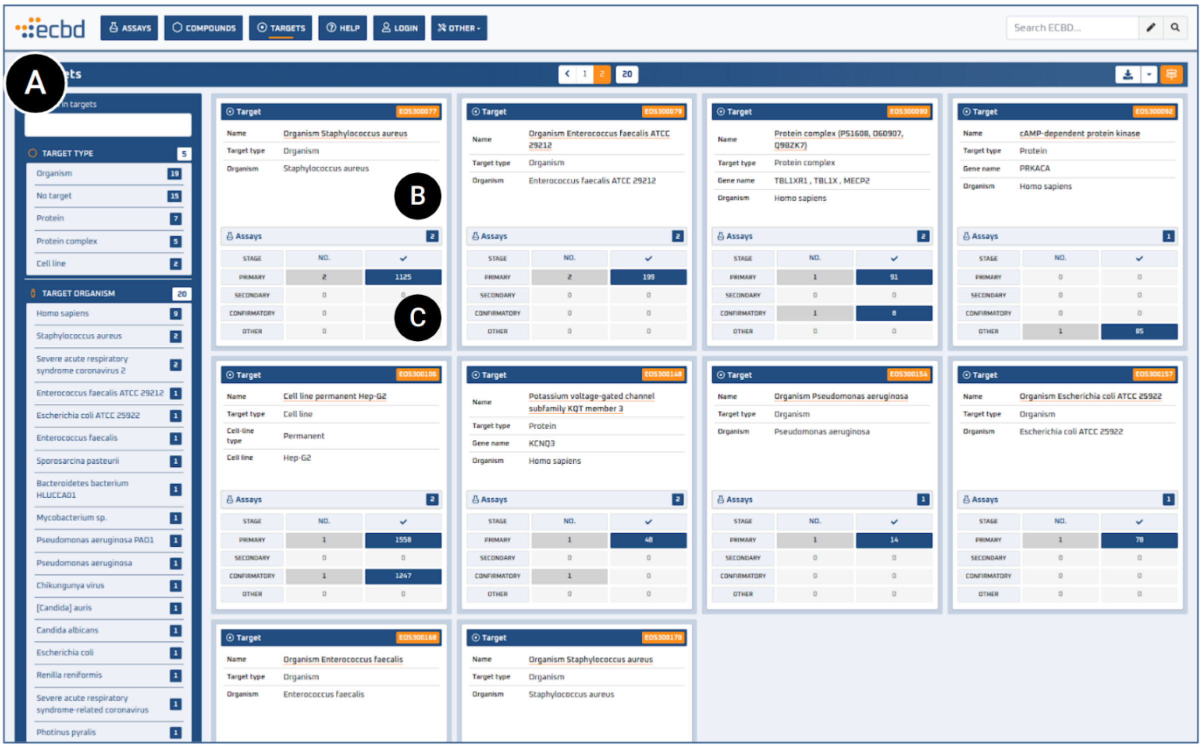

Figure 4. Target browse view with the left navigation bar (A) and the target representation divided into 2 sections (B and C). A) The navigation. B) The Target's general information - EOS ID, name, type and organism. C) The assay summary table containing compounds and their results.

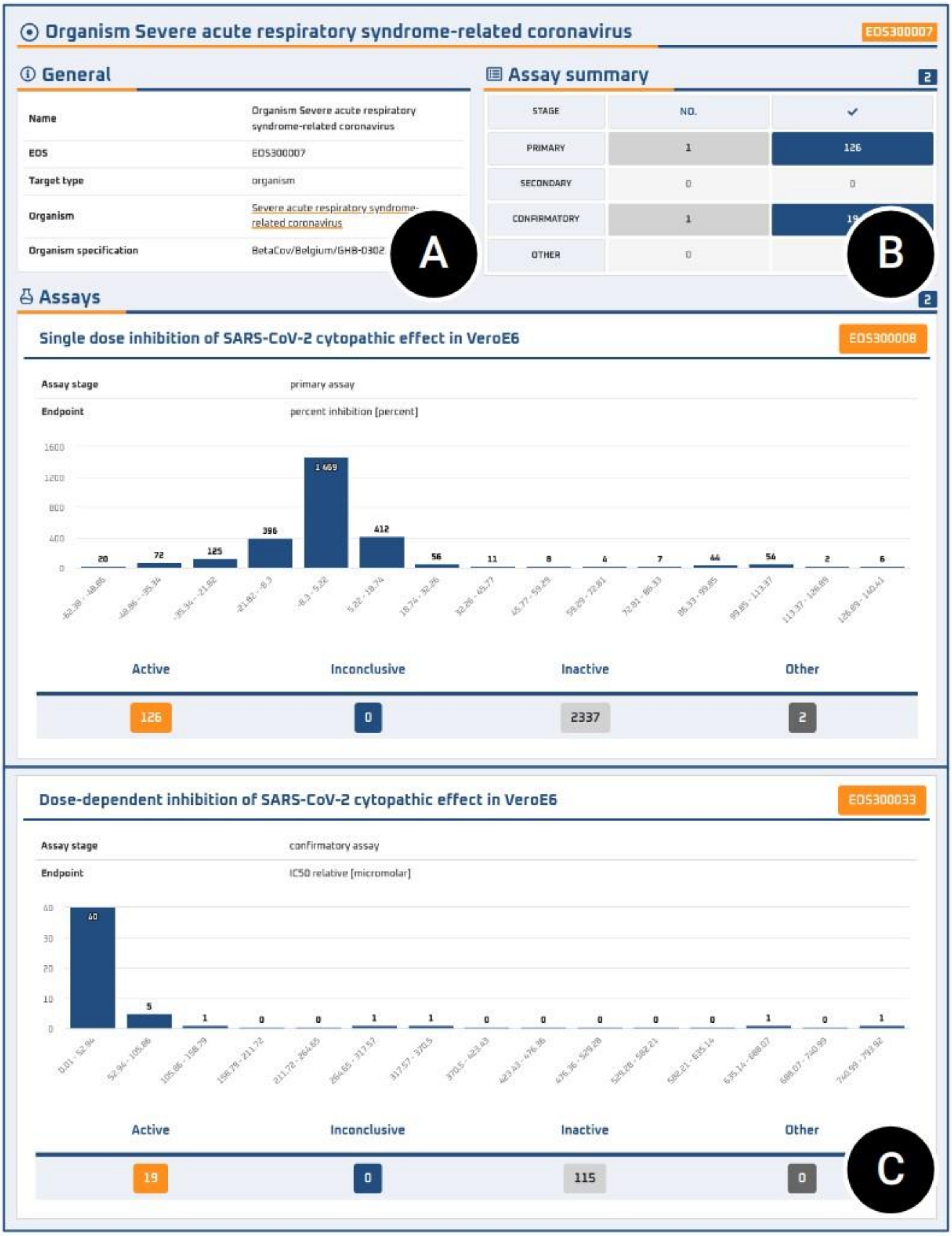

Figure 5. Target detail view containing (from top to bottom). A) General information including the name, EOS ID, type, organism and its specification. B) The assay summary table containing compounds and their results. C) The activity distributions within different assays.

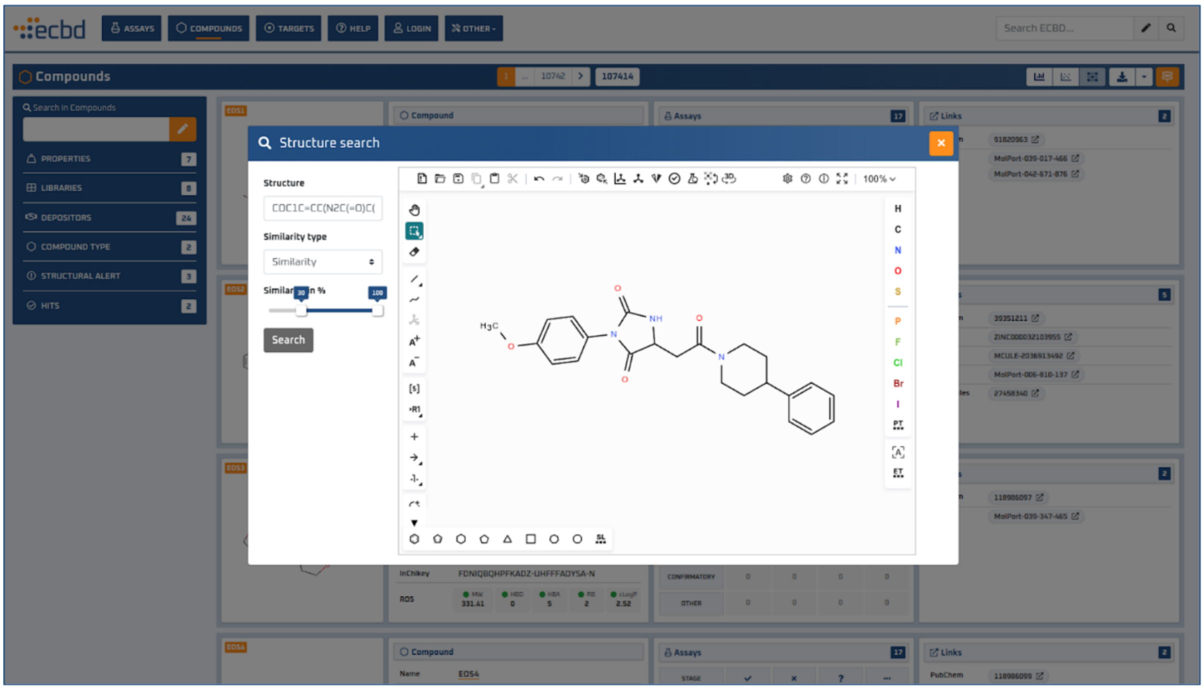

Figure 6. The chemical structure editor, Ketcher.

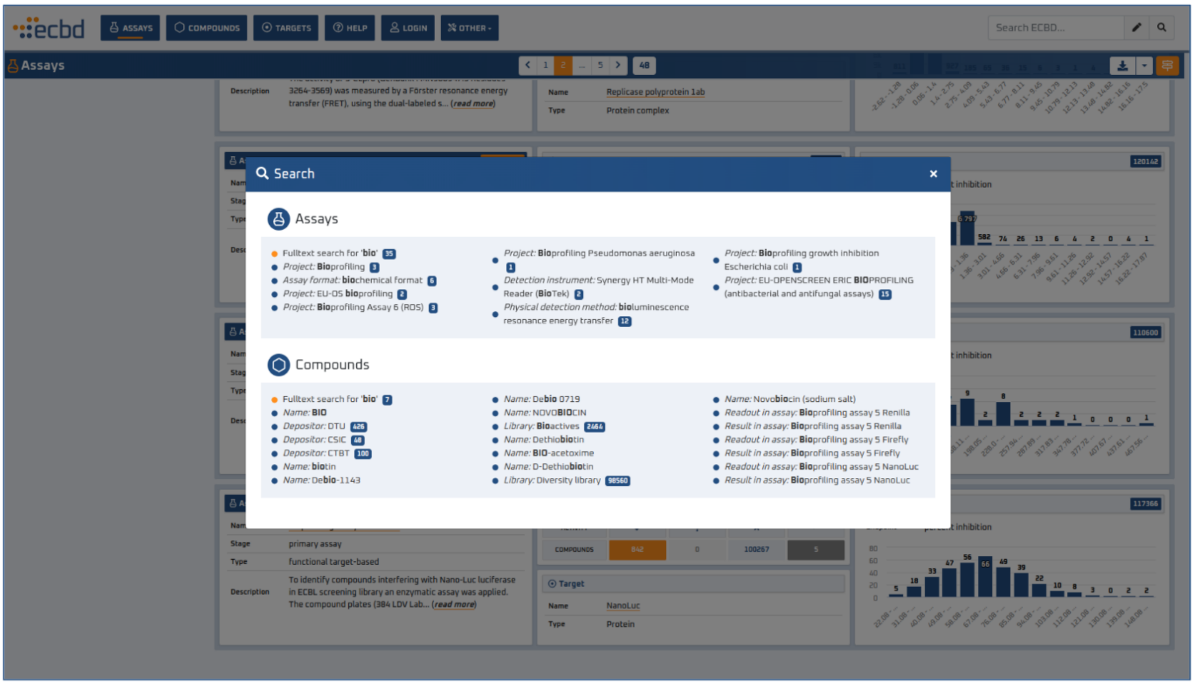

Figure 7. Text search result. Specific results are divided into categories based on their data type.

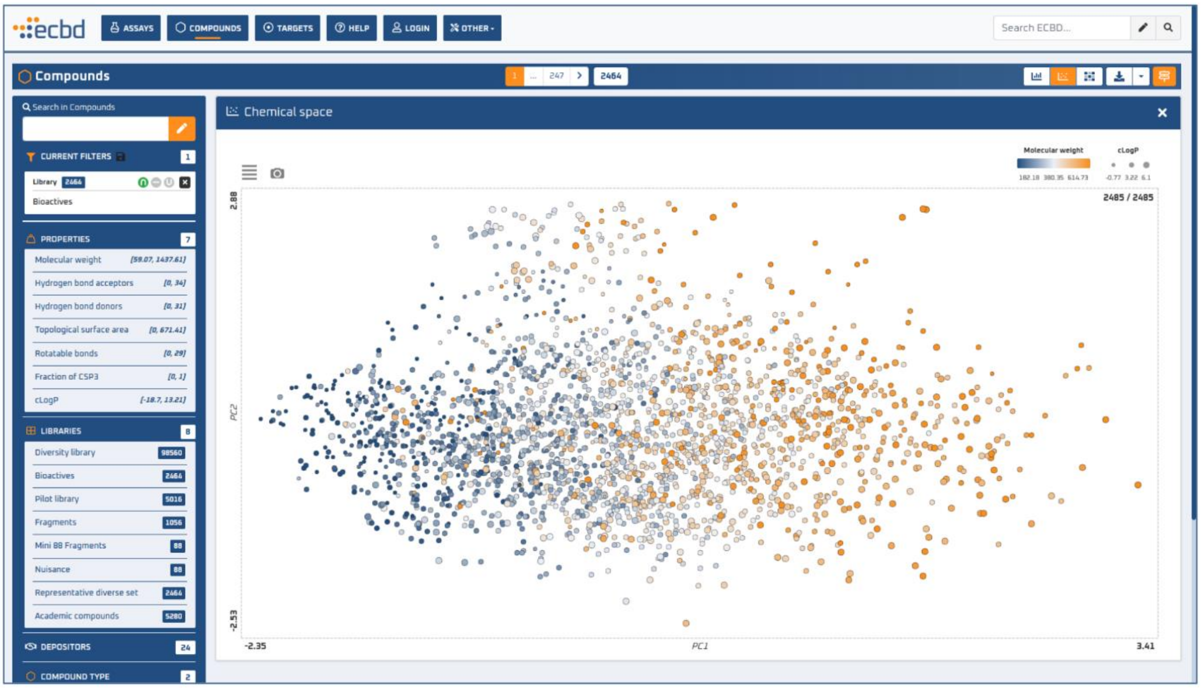

Figure 8. Chemical space (PCA based on the ECFP4 fingerprint) of the currently selected compound set (here for the bioactive compound library). The colour and size of the compounds (points) depend on their physicochemical properties (here, the colour on their molecular weight and the point size on their lipophilicity - calculated logP).

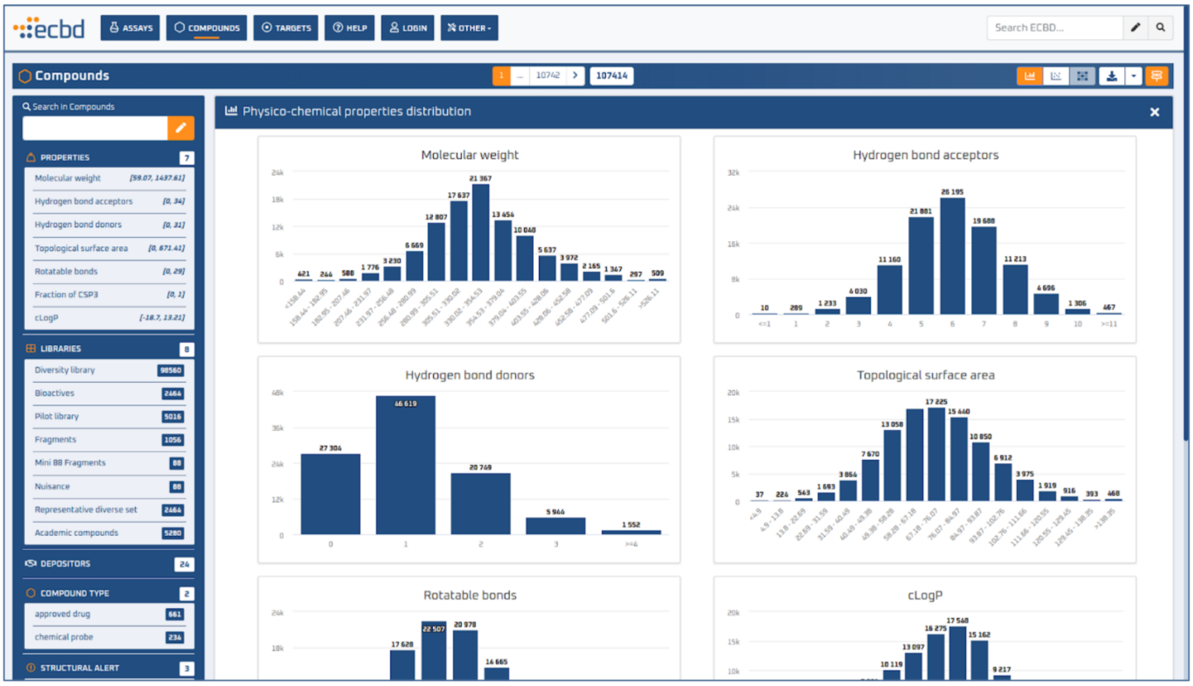

Figure 9. Bar chart distribution of compounds' physicochemical properties.

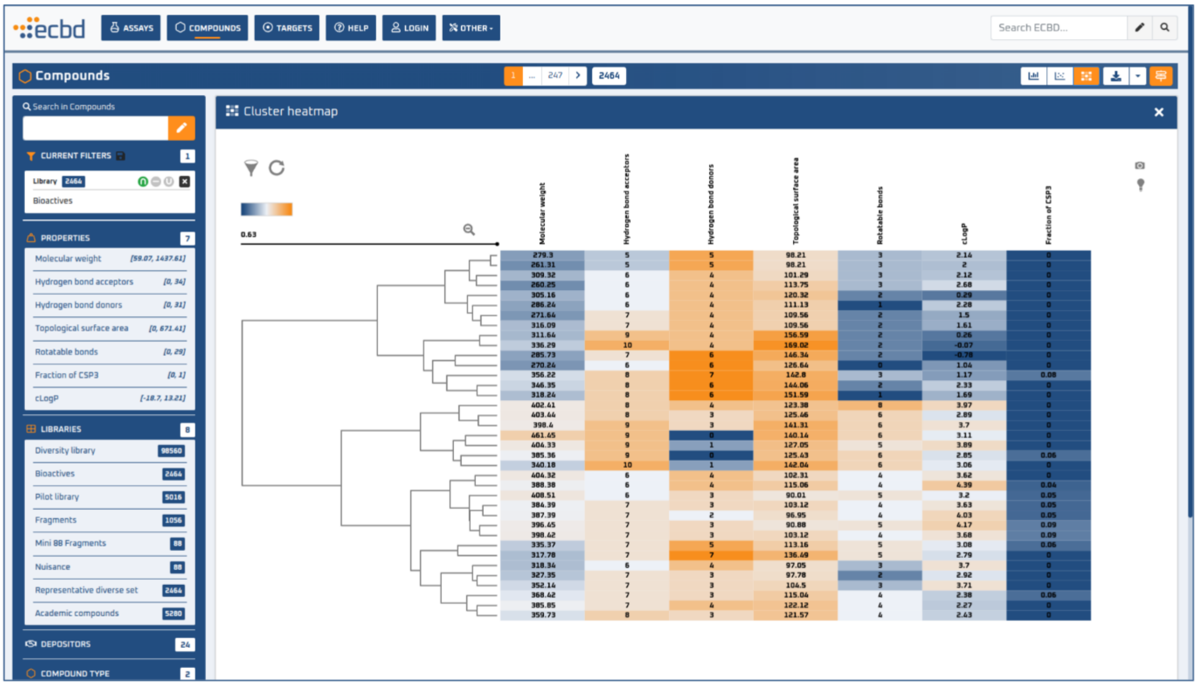

Figure 10. Cluster heatmap of a selected compound set (the bioactive library). The compounds are clustered based on their physicochemical properties using Euclidean distance and Ward's linkage.

API

ECBD API (Application Programming Interface) is based on the Django REST framework (https://www.django-rest-framework.org/). API provides access to the data stored in the database in JSON format without the need to use the web interface or to download the database dump. The API entry point is at https://ecbd.eu/api/ and it contains links to 3 subsections - ECBD's main data types (assays, compounds and targets). Each link leads to the paginated list of given objects (e.g. https://ecbd.eu/api/compounds/ leads to the first 100 compounds), the next page can be accessed with the URL contained in the "next" parameter of the response (e.g. https://ecbd.eu/api/compounds/?limit=100&offset=100"). All information about a specific object can be accessed by its EOS identifier and include=all parameter in the URL request (e.g. https://ecbd.eu/api/assays/EOS300008/?include=all).

API examples

Example ***https://ecbd.eu/api/compounds/?limit=100&offset=100"***


```
{
    "count": 107448,
    "next": "https://ecbd.eu/api/compounds/?limit=100&offset=200",
    "previous": "https://ecbd.eu/api/compounds/?limit=100",
    "results": [
        {
            "id": 101,
            "pk": 101,
            "_label": "Cc1nc(NC(=O)CC[C@@H]2NC(=O)N(Cc3ccc4c(c3)OCO4)C2=O)sc1C",
            "cls": "core | compound",
            "eos": "EOS101",
            "attributes": {
                "name": [],
                "cas": [],
                "tag": [],
                "attribute": [],
                "text": []
```

```
        },
        "assay_summary": {
            "primary": {
                "active": 0,
                "inconclusive": 0,
                "inactive": 16,
                "other": 1
            },
            "secondary": {
                "active": 0,
                "inconclusive": 0,
                "inactive": 0,
                "other": 0
            },
            "confirmatory": {
                "active": 0,
                "inconclusive": 0,
                "inactive": 0,
                "other": 0
            },
            "other": {
                "active": 0,
                "inconclusive": 0,
                "inactive": 0,
                "other": 0
            },
            "all": 17
        },
        "depositor_compoundid": null
    },
    ….
]
```

}

Example https://ecbd.eu/api/compounds/EOS101/?include=all


```
{
    "id": 101,
    "pk": 101,
    "_label": "Cc1nc(NC(=O)CC[C@@H]2NC(=O)N(Cc3ccc4c(c3)OCO4)C2=O)sc1C",
    "cls": "core | compound",
    "structure": {
        "id": 300100,
        "pk": 300100,
        "svg": "/static/compounds/svg/default/MNARCEZQAVKCAR-ZDUSSCGKSA-N.svg",
        "violates_ro5": 0,
        "links": [
            [
                {
                    "ligand_id": "75489923",
                    "db": "PubChem",
                    "url": "https://pubchem.ncbi.nlm.nih.gov/compound/75489923"
                }
            ],
            [
                {
                    "ligand_id": "ZINC000096231535",
                    "db": "ZINC",
                    "url":
"http://zinc15.docking.org/substances/ZINC000096231535/"
                }
            ],
            [
                {
                    "ligand_id": "MolPort-029-883-745",
```

```
                    "db": "MolPort",
                    "url": "https://www.molport.com/shop/molecule-link/MolPort-029-883-745"
                }
            ]
        ],
        "name": "",
        "smiles": "Cc1nc(NC(=O)CC[C@@H]2NC(=O)N(Cc3ccc4c(c3)OCO4)C2=O)sc1C",
        "molblock": "", // molfile from the imported sdf
        "inchi": "InChI=1S/C19H20N4O5S/c1-10-11(2)29-18(20-10)22-16(24)6-4-13-17(25)23(19(26)21-13)8-12-3-5-14-15(7-12)28-9-27-14/h3,5,7,13H,4,6,8-9H2,1-2H3,(H,21,26)(H,20,22,24)/t13-/m0/s1",
        "inchikey": "MNARCEZQAVKCAR-ZDUSSCGKSA-N",
        "formula": "C19H20N4O5S",
        "mw": 416.4590148925781,
        "hba": 9,
        "hbd": 2,
        "tpsa": 109.86000061035156,
        "rb": 6,
        "fp3": 0.3684210479259491,
        "logp": 2.327939987182617,
        "description": ""
    },
    "eos": "EOS101",
    "libraries": [
        {
            "name": "European Chemical Biology Library",
            "pk": 1
        },
        {
            "name": "ECBL Pilot Compounds",
            "pk": 4
        },
```

```
        {
            "name": "Representative set of the diversity library",
            "pk": 9
        }
    ],
    "api_url": "https://ecbd.eu/api/compounds/EOS101/",
    "attributes": {
        "name": [],
        "cas": [],
        "tag": [],
        "attribute": [],
        "text": []
    },
    ....
}
```

Example [*https://ecbd.eu/api/assays/EOS300008/?include=all*](https://ecbd.eu/api/assays/EOS300008/?include=all)

```
{
    "id": 300008,
    "pk": 300008,
    "_label": "Assay object (300008)",
    "intended_target": {
        "id": 300007,
        "pk": 300007,
        "_label": "Organism Severe acute respiratory syndrome-related
coronavirus",
        "cls": "core | target",
        "type": "Organism",
        "gen_name": "Organism Severe acute respiratory syndrome-related
coronavirus",
        "eos": "EOS300007",
        "target_type": "organism",
        "nucleicacidtype": "DNA",
```

"sequence": "",
"celline_type": "permanent",
"organism_specification": "BetaCov/Belgium/GHB-03021/2020",
"target_fingerprint": "c953f29c7344427f23b26c31a76af60a",
"name": "Organism Severe acute respiratory syndrome-related coronavirus"
},
"cls": "core | assay",
"eos": "EOS300008",
"api_url": "https://ecbd.eu/api/assays/EOS300008/",
"activities": {
"summary": {
"active": 126,
"inactive": 2337,
"inconclusive": 0,
"other": 2
},
"compounds": 2464,
"endpoint": {
"id": 2,
"endpoint_ontology": "bao",
"endpoint_iri": "http://www.bioassayontology.org/bao#BAO_0000201",
"endpoint_label": "percent inhibition",
"unit_ontology": "bao",
"unit_iri": "http://purl.obolibrary.org/obo/UO_0000187",
"unit_label": "percent"
},
"values": {
"count": 2686,
"x_min": -62.38,
"x_max": 140.41,
"y_min": 2.0,
"y_max": 1469.0,

"data": [
    20.0,
    72.0,
    125.0,
    396.0,
    1469.0,
    412.0,
    56.0,
    11.0,
    8.0,
    4.0,
    7.0,
    44.0,
    54.0,
    2.0,
    6.0
],
"labels": [
    "-62.38 - -48.86",
    "-48.86 - -35.34",
    "-35.34 - -21.82",
    "-21.82 - -8.3",
    "-8.3 - 5.22",
    "5.22 - 18.74",
    "18.74 - 32.26",
    "32.26 - 45.77",
    "45.77 - 59.29",
    "59.29 - 72.81",
    "72.81 - 86.33",
    "86.33 - 99.85",
    "99.85 - 113.37",
    "113.37 - 126.89",

```
            "126.89 - 140.41"
        ]
    }
},
"endpoints": [
    {
        "id": 2,
        "endpoint_ontology": "bao",
        "endpoint_iri": "http://www.bioassayontology.org/bao#BAO_0000201",
        "endpoint_label": "percent inhibition",
        "unit_ontology": "bao",
        "unit_iri": "http://purl.obolibrary.org/obo/UO_0000187",
        "unit_label": "percent"
    },
    {
        "id": 1,
        "endpoint_ontology": null,
        "endpoint_iri": null,
        "endpoint_label": "percent confluence",
        "unit_ontology": "bao",
        "unit_iri": "http://purl.obolibrary.org/obo/UO_0000187",
        "unit_label": "percent"
    }
],
"results": {
    "CONTROL-1": {
        "type": "low",
        "values": [
            {
                "endpoint_id": 1,
                "value": -0.01,
                "operator": "EQ",
```

```
                "concentration": 10.0,
                "time": null,
                "comment": "",
                "plate": "ESP0025890"
            },
            {
                "endpoint_id": 1,
                "value": 0.08,
                "operator": "EQ",
                "concentration": 10.0,
                "time": null,
                "comment": "",
                "plate": "ESP0025890"
            },
        ....
}
```
