# Supplementary material for: ECBD: European chemical biology database
Source: Nucleic Acids Res. 2024 Oct 23;53(D1):D1383–92. doi: 10.1093/nar/gkae904 (PMC11701612; doi:10.1093/nar/gkae904)
Supplement: gkae904_Supplemental_Files [file gkae904_supplemental_files.zip › supplementary_file_2.pdf]

HIGH-THROUGHTPUT SCREENING QUALITY CONTROL GENERAL GUIDELINES

EU-OPENSCREEN ERIC (EU-OS), the European Research Infrastructure Consortium of Open Screening Platforms for Chemical Biology, builds a distributed organization of national screening and chemistry facilities, a common database, and a central headquarter that manages a joint compound collection and coordinates project flow and training. It provides world-class services to academia and industry in the fields of small-molecule screening and medicinal chemistry.

To ensure that data produced at different sites are comparable and reproducible, a set of common operational standards for the EU-OPENSCREEN screening sites have been defined. While the operational standards need to cover several aspects of the screening, they are here kept to a minimum in order to avoid becoming a burden to the screening sites.
As a general rule, assays that cannot meet more than 3 of the reported quality criteria shall be discussed with and approved by EU-OPENSCREEN Central Office.

The following High-throughput screening (HTS) guidelines are based on the "Report on screening standards to be used" (Deliverable 11.7, October 2013, Grant Agreement number: 261861) and regulate the following aspects:

1. Library reformatting and delivery

As reported in the "Guidelines for use of the EUROPEAN CHEMICAL BIOLOGY LIBRARY (ECBL)", compounds are reformatted and provided to the Screening Partner Sites (SPSs) as summarized below.

- Central Compound Management Facility (CCMF) support for High-Capacity Screening Sites (HCSS): HCSS will receive 30 µL of the 10 mM solution in 384-well plates (3x9 µL when Low Dead Volume (LDV) plates are requested). HCSS may further aliquot or process the aliquot according to individual requirements. This amount shall be sufficient to generate assay plates for about 10 primary screening campaigns (9 primary screening campaigns when LDV plates have been requested), and to conduct cherry picking/IC50 validation experiments. Depending on overall usage, it is anticipated that resupply of the entire ECBL will be provided to the HCSS by the CCMF after usage of the compound library in these screens. If a site runs out of volume, resupply volume is individually negotiated based on the planned number of

screens in the future. Upon request, the CCMF will provide cherry pick samples of 5 µL of a 10 mM solution for up to 500 hit compounds per HTS project for follow-up experiments, or up to 25 compounds if only the pilot library is screened.

- CCMF-support for Specialist Screening Sites (SSS) and Assay-Adaptation Sites (AAS): SSS will receive up to 30 µL of the 10 mM solution of the ECBL-Pilot (15 plates x 352 compounds, last two columns empty reserved for positive and negative controls) in 384-well plates from the CCMF. SSS may further aliquot or process the aliquot according to individual requirements. This amount shall be sufficient to generate assay plates for primary screening and to conduct cherry picking/IC50 validation experiments. Resupply of the mother plates will be provided to the SSS by the CCMF if at least 10 screens have been implemented (9 screens when LDV plates have been requested). For cherry picks from the pilot screen library, the CCMF will provide 5 µL of the 10 mM stock solution for up to 25 compounds per project. SSS can also request 30 µL of the 10 mM solution of the ECBL if correct storage can be ensured. In this case rules described for HCCS applies. Moreover, SSS can request the entire ECBL in assay-ready plates on a project-by-project basis. Assay-ready plates will be provided directly from the CCMF.
- The central compound management facility performs quality control of the compounds.
- The standard 384-well plate layout format is as follow: 352 compounds per plate, distributed in columns 1 to 22. The last two columns (column 23 and 24) are left empty for running controls during the screening. Different plate layout formats need to be discussed with and approved by the CCMF.
- An overview of the compounds provided to the Partner Sites is available on sharepoint and constantly updated by the CCMF team.

2. Compound storage at Screening Partner Site

- Assay ready plates will be stored sealed at -20°C, below 25% of humidity condition. Upon thawing, assay ready plates are used immediately as a whole batch. Storage time of assay ready plates is not limited but it will always be recorded and kept to minimum.
- Stock plates will be stored sealed at -20°C, below 25% of humidity condition. Stock plates may undergo maximally 10 freeze-thaw cycles. Storage time of stock plates is kept to a minimum and the duration will always be recorded. The number of freeze-thaw cycles of stock plates and liquid handling i.e. number of transfers from the stock plates, are always recorded.
- Different storage conditions must be discussed with and approved by EU-OPENSCREEN Central Office.
- If any precipitation occurs in the stock plates at the beginning/during freezing/thawing, it is documented, but no further action should be applied.

3. **Robotics and automation**
   - EU-OPENSCREEN screening site will develop and follow Standard Operating Procedures (SOPs) for operating instruments.
   - EU-OPENSCREEN screening site will perform quality analysis according to instrument manufacturers' advice and perform assay-specific quality control of equipment before starting a screening project.

4. **Assay development and follow up plan after primary screening**
   - EU-OPENSCREEN screening site will offer assistance in assay development and/or put the project owner in contact with a suitable assay development site.
   - A detailed follow-up plan will be required, including description of the assays, and refined between the screening centre and the project owner before starting the screen.
   - In case that secondary assays will be performed by the SPS, it will be required that the follow-up assays are optimised and validated at the screening site before starting the screening.

5. **Assay quality criteria**
   a. **Reagents quality control**
      - Biochemical reagents
        For biochemical assays, reagents (e.g. proteins, enzymes, buffers) shall be stable in the experimental conditions tested for the entire duration of the run using these reagents. Signal stability should be proven before the beginning of the experiment.
      - Cell line quality control
        Ideally, if not obtained directly from the supplier quality and integrity of cell lines used for drug screening should be proven through short tandem repeat (STR) profiling (https://www.atcc.org/en/Products/Cells_and_Microorganisms/Testing_and_Characterization/STR_Profiling_Analysis.aspx).
   b. **Intra-plate quality control**
      - **DMSO Tolerance**
        DMSO related assay sensitivity should be determined before starting the assay. Minimum DMSO tolerance is defined as the DMSO concentration that does not negatively affect assay readout (i.e. enzyme activity or cell viability) in terms of signal variation from wells without DMSO. Signal should not deviate more than 20% from signal without DMSO. As a general rule, the assay system should at least tolerate 1% DMSO for biochemical screens and at least 0.1% for cell-based screens.
      - **Z'-factor and robust Z'-factor**

Minimum Z´ factor cut-off (Z´ > 0.5) (Zhang JH, 1999) is applied per default for reader-based biochemical and cellular screens. A 0.4 < Z' < 0.5 can be accepted for cellular assays if the total number of hits can be validated in follow-up assays. This has to be demonstrated by screening the pilot library. When Z´ cut-off is not applicable, an alternative method to assess whether the response in an assay is significant can be defined. The alternative parameter has to be defined up front and approved by EU-OPENSCREEN. If well-argued and accepted by EU-OPENSCREEN, plates with Z' < 0.5 could be used as long as this is registered in the experimental report.

The use of robust *Z′*-factor where standard deviation is replaced by robust standard deviation [median absolute deviation (MAD)×1.483] and mean by median in the Z'-factor equation is allowed to remove the influence of outliers (Murray D, 2016).

- **Coefficient of variation**

  When Z'-factor cannot be applied, data variability will be reported instead using the coefficient of variation (CV%). CV% refers to the sample population of a screening plate. CV% must be lower than 20%.

- **Minimum Significant Difference**

  Priority will be given to Z'-factor and CV%. When a sufficient number and range of XC50 values can be measured, Minimum Significant Difference (MSD) will be used as additional parameter for assessing assay reliability. MSD represents the smallest efficacy difference between two compounds that is statistically significant (*https://www.ncbi.nlm.nih.gov/books/NBK83783/*). MSD lower than 20 is suggested. If the use of MSD is not applicable, inter-day variation CV may be used instead. At least three replicas in three independent days should be carried out in order to check inter-day variability.

6. Pilot screen

A pilot screen with a sub-library comprising 5016 compounds (2464 bioactives, 2464 representatives of the ECBL and 88 assay interfering compounds, the latter plated at four different concentrations) is performed once to validate assay performance and robustness. The pilot screen serves to establish the assay protocol, data evaluation, estimation of hit rate and test for robustness of the assay against compound-induced measurement artifacts (as frequently caused by cytotoxicity, auto-fluorescence or aggregation).

- **Positive and negative controls**

  When available, the following number of controls will be used:

  - 8 controls/ 96-well plate
  - 32 controls/ 384-well plate
  - 64 controls/ 1536-well plate

Negative controls must consist of the reactions with no compound (DMSO only) and must be present on each testing plate. Ideally, reference compounds are used for generating the positive control. Only in the absence of any known compound standards or if the effect on assay readout is unstable, other measures for generating the positive controls are acceptable (e.g. knock-out cell lines for cellular assays, or wells without enzyme for biochemical assays).

- **Hit-rate**
  The acceptable range for the hit rate will be defined at the beginning of each screening campaign in agreement between user and screening site. Whenever possible a counter-screen should be identified and in case of elevated hit rates, the screening site may insist in the establishment of a counter-screening protocol.
  In the case of very low hit rates the screening campaign is stopped (assay sensitivity too low). In the case of high hit rates (assay specificity too low) the compound concentration can be reduced or the activity threshold for a hit can be changed.

7. Failure rate

After initial quality control, no more than 20% of the measured plates shall have to be remeasured. An initially failed plate (excluding technical failures) can be repeated, but to a maximum of 3 repetitions.

8. Data analysis

Per default, primary screens, hit validation and data analysis must be performed by the EU-OPENSCREEN ERIC Screening Partner Site to ensure sufficient quality of data which will be uploaded into the ECBD. Exceptions, such as the joint generation of raw data and its analysis between a partner site and an external user laboratory, might be acceptable only in exceptional circumstances, when specific technical requirements (such as higher safety levels required, shifts to a different laboratory due to Covid-19-related restrictions) are necessary for the implementation of the screening assay. In such cases, the external user laboratory must follow the HTS guidelines and the involved EU-OPENSCREEN screening partner site is fully responsible for the quality of the data which is uploaded into the ECBD. Exceptions must be discussed with and need to be approved by the EU-OPENSCREEN ERIC Central Office. No primary screen can be performed without involvement of an accredited EU-OPENSCREEN Screening Partner Site.

9. Correction of plate patterns and outliers

- Spatial patterns correction algorithms (like b-scores) are not recommended. Hardware-based correction for spatial patterns might be acceptable (e.g. the flatfield-correction from Perkin Elmer Envision system). Intra-plate normalisation to reduce plate patterns will be

allowed only in special types of assays/ readouts and it should be justified before the screening campaign begins and approved by EU-OPENSCREEN

- Outlier correction will not be allowed for single dose assays. All data have to be uploaded into the ECBD and if necessary flagged as “inconclusive”.
  Regarding control wells, outliers (3 σ) can be removed as follow: up to 1 positive OR 1 negative control in the 96 well plate format, up to 2 positive AND 2 negative controls in the 384-well plate format and up to 4 positive and 4 negative controls in the 1536-well format (or in general, 1/8 of the control wells). However, reasonable activity ranges can be applied for hit definitions (e.g. no activities below negative controls) when creating the activity-call column for the ECBD database upload.
  Outlier correction is allowed for dose response assays. Outlier values removed for XC50 calculation should be uploaded together with the accepted data points, and correspondingly be flagged as outliers.

10. Documentation of screening procedure

- English language will be used for all documentation related to EU-OPENSCREEN projects.
- Operational and instrument running logs will be recorded and stored at the screening site.
- EU-OPENSCREEN screening sites will ensure that operational logs are written by the person performing the screen and that a copy is stored at the screening site for the exceptional cases described in paragraph 8.
- Information on reagents (supplier and batch number) will be recorded and stored at the screening site and in the ECBD.
- EU-OPENSCREEN highly recommends the screening sites to use LIMS locally.
- Assay descriptions will be recorded as follow:

DATA FIELD	STANDARD OR ALLOWED VALUES	MORE INFORMATION/ EXAMPLES
**Assay Title**	As per depositor internal standard	Must be comprehensive and informative
**Project**	As per depositor internal standard	Must be comprehensive and informative. Connects related assays (e.g., primary and confirmatory assays)
**Description**	Free text	Must be comprehensive and informative
**Assay type**	BioAssay Ontology, select from descendents of BAO_0000008 (bioassay type)	E.g. BAO_0000041 (binding)

**Assay setting**	BioAssay Ontology, select from descendents of BAO_0020005 (experimental setting)	E.g. BAO_0020008 (in vitro)
**Assay stage**	BioAssay Ontology, select from descendents of BAO_00000029 (assay screening campaign stage)	E.g. BAO_0000031 (primary assay)
**Assay design**	BioAssay Ontology, select from descendents of BAO_0002202 (bioassay method)	E.g. BAO_0002755 (fluorescent ligand binding method)
**Physical detection method**	BioAssay Ontology, select from descendents of BAO_0000035 (physical detection method)	E.g. BAO_0000005 (flow cytometry)
**Detection instrument**	BioAssay Ontology, select from descendents of BAO_0000697 (detection instrument)	E.g. BAO_0000701 (EnVision Multilabel Reader)
**Assay support**	BioAssay Ontology, select from descendents of BAO_0002429 (assay supporting method)	E.g. BAO_0002479 (photoaffinity labeling)
**Cellular component**	Gene Ontology, select from descendants of GO_0005575 (cellular component)	E.g. GO_0005737 (cytoplasm)
**Organism**	NCBI taxonomy	E.g. 9606 (Homo sapiens)
**Target type**	Select from list: protein, protein complex, cell line, nucleic acid, organism, tissue, pathway, no target	E.g. protein
**Intended target**	Depending on the Target type: UniProt/ChEMBL for protein/protein complex; Cellosaurus ontology for cell line; NCBI taxonomy for organism; List selection from DNA, RNA, pre-mRNA, tRNA, rRNA for nucleic acid + organism; Brenda ontology for tissue; Reactome Ontology for pathway;	E.g. P04150 (Glucocorticoid receptor, uniPort), DNA/9606 (Homo sapiens, NCBI taxonomy)

**Concentration unit**	Units of measurement ontology, select from descendants of UO_0000051 (concentration unit)	E.g. UO_0000063 (millimolar)
**Time unit**	Units of measurement ontology, select from descendants of UO_0000003 (time unit)	E.g. UO_0000032 (hour)
**Endpoint**	BioAssay Ontology, select from descendents of BAO_0000179 (result)	E.g. BAO_0000656 (efficacy)
**Unit**	BioAssay Ontology, select from descendents of BAO_0000077 (unit of measurement)	E.g. BAO_0080022 (log change)

- In case any of the assay description fields are not relevant/applicable to the assay, they can be tagged as *Not applicable*.
- In case the data uploader (EU-OPENSCREEN screening site) can't find a proper term to describe the assay in the used ontology, any custom value can be used instead. EU-OPENSCREEN database site will process these terms and integrate them in ECBD (eventually will attempt to propagate these terms to the employed ontologies).

11. Formulas and definitions

a. The parameters for Z prime calculation are the standard deviations of the positive (σp) and negative (σn), and means of the positive (µp) and negative (µn) controls.

The equation: **Z´ factor** = $1 - (3(\sigma^p + \sigma^n))/(|\mu^p - \mu^n|)$

The equation for **standard deviation**: $\sigma = \sqrt{\frac{\Sigma(x-\mu)2}{N}}$, where σ = standard deviation, x = each value in the population, μ = mean of the values, N = number of values

b. **Coefficient of variation:** $CV = \frac{\sigma}{\mu}$

c. **Single dose active compound**: average plus/minus three standard deviations. Special assays might benefit from deviating criteria. Alternative criteria have to be defined up front and approved by the screening site.

d. **XC50**= half maximal response concentration (IC50, EC50 or AC50)

e. **Dose response active compound**: an XC50 value lower than the highest tested dose

f. **XC50 values will be calculated according to the following definitions:**

- Minimum of 8 data points (concentrations) are used for calculating an XC50.
- Minimum of 2 replicas for each experimental data will be used.
- Dose response curve fitting is done using the four-parameter logistic model as default.

$$y = Bot + \frac{Top - Bot}{1 + 10^{(\log_{10}(IC50) - \log_{10}(x)) * slope}}$$

Bot: minimal response

Top: maximal response

IC50: concentration of drug at the symmetric inflection point

slope: slope of the tangent to the curve when the concentration is IC50 (Hill-coefficient).

x: concentration

y: response

If the positions of IC50 and x are interchanged in the formula, the sign of the slope is changed.

For each assay, limitations of parameters are specified.

- An XC50 must be within the tested range - no extrapolation is allowed. XC50s outside the tested range are specified as: >[highest tested dose] (inactive) or "<[lowest tested dose]"
- XC50 error will be reported as error range (95% confidence interval) and fitting quality using R square.
- An XC50 must have a minimum visible activity change (activity difference between highest and lowest tested dose, determined using the fitted curve) of at least 25% in case of IC50. In case of EC50, this value has to be defined in the biological context of the assay. 25% is a minimal visible activity change, but other values >25% can be set for specific experiments and reported along with EC50 data.

EU-OPENSCREEN ERIC COMPOUND COLLECTION (EU-OS ERIC COMPOUND COLLECTION) is a screening collection comprising COMMERCIAL COMPOUNDS and/or ACADEMIC COMPOUNDS.

SCREENING PARTNER SITES (SPS) are the research institutes that provide the respective experimental facilities for compound screening and bioprofiling of compounds contained in the EU-OPENSCREEN COMPOUND COLLECTION. SPS are categorized in High-Capacity Screening Sites (HCSS) and Specialized Screening Sites (SSS). HCSS will receive and host compounds from the EU-OPENSCREEN ERIC compound collection. Typically, one high capacity screening site within each country will act as the national repository and distribute the EU-OPENSCREEN ERIC collection compounds to other partner sites (e.g. specialised sites) within that country. SSS will add expertise and technology to the EU-OPENSCREEN ERIC that is not commonly offered by screening facilities (e.g. BSL-3 capacities, radioactivity, screening in 3D cellular models etc.)

ASSAY-ADAPTATION SITES (AAS) are sites that, in close collaboration with users, will adapt bench-top protocols into high-throughput screening (HTS) format and optimise the assay performance under automated or semi-automated screening conditions. Typically, validated assays will then be transferred to specialist or high capacity screening sites. Assay adaptation sites may also perform compound screening on small to medium scales.

The **EUROPEAN CHEMICAL BIOLOGY DATABASE (ECBD)** is EU-OPENSCREEN ERIC's open access database, in which structural information of commercial and proprietary compounds, bioprofiling results and primary screening data will be published.

USER shall mean any individual or its authorized representative, any legal entity or any organization utilizing COMPOUNDS for screening purposes through the SCREENING PARTNER SITE(S).

ASSAY(s) shall mean bioassay(s) in which a procedure is carried out containing experiments for determining the biological activity of COMPOUND(s) by measuring one or multiple effect(s) on a biomolecule, an organism, a tissue, a cell line or a biological model compared to control compounds.

PRIMARY ASSAY is the first assay performed in the screening campaign. The purpose of the primary assay is to identify primary hits, which are potentially biologically active chemical entities.

SECONDARY ASSAYS are the additional assays following the hit validation stage to confirm the biological activity of chemical entities via a different type of assay or to eliminate certain active compounds based on their mechanism of action, toxicity or activity profile. SECONDARY ASSAYS can also include selectivity and specificity assays.

CONFIRMED HITS are compounds which were initially identified as hits in the primary assay and then retested at the same concentration (ideally in at least duplicates) in the same assay in order to exclude technical false positives.

COUNTER ASSAY is the assay run to eliminate those hits from the primary and confirmatory assay stages that are not of interest due to their artificial or non-selective activity

ASSAY READY PLATE is the screening plate containing a small aliquot of the compound to be screened, sufficient for a single ASSAY.

VALIDATED HIT is a term for a putative biological activity of a compound, validated by a dose-response curve and usually expressed as an EC50 (for stabilizers and activators) or an IC50 (for inhibitors) value. This is accompanied by lack of activity in assay-relevant counter screens and by independent confirmation of compound purity and identity.

DATA shall mean information output by any sensing device. In this present contract, it defines all information pertaining the physical and chemical property of a compound (for instance, mass spectra trace, molecular weight, etc.) and all physical-chemical or biological information originates from a) bioprofiling assessment and b) screening assays.
